# Analysis of 515 *human* proteomes defines plasma extracellular vesicle core composition, corona and coisolated networks

**DOI:** 10.1038/s42003-026-11038-6

**Published:** 2026-10-30

**Authors:** Ana Sofia Carvalho, Juan M. Falcón-Pérez, Hans Christian Beck, Rune Matthiesen

**Affiliations:** 1 iNOVA4Health, NOVA Medical School, Faculdade de Ciências Médicas, Universidade NOVA de Lisboa, Lisboa, Portugal; 2 Exosomes Laboratory. CIC-bioGUNE-BRTA—Centro de Investigación Cooperativa en Biociencias, Vizcaya, Spain; 3 IKERBASQUE Research Foundation, Bilbao, Spain; 4 Centre for Clinical Proteomics, Department of Clinical Biochemistry, Odense University Hospital, Odense, Denmark

**Keywords:** Systems analysis, Biomarkers, Proteomics

## Abstract

Plasma extracellular vesicles (EVs) are promising liquid biopsy biomarkers, yet their proteomic profiling remains confounded by massive co-isolation. Here, we resolve this heterogeneity through a meta-analysis of 515 *human* plasma EV proteomes. We demonstrate that the reproducible core proteome is overwhelmingly governed by predictable, surface-charge-driven mass-action that dynamically mirrors synthetic nanoparticle corona formation. By quantitatively decomposing the core isolate against pan-synthetic coronas and agonist-induced platelet EVs, we trace the core isolate to three main mechanistic origins: an electrostatic plasma corona, ex vivo platelet vesiculation and biological macromolecular co-isolates. Crucially, the residual vesicular cargo strongly correlates with thrombin-induced platelet degranulation, indicating that phlebotomy-induced trauma contribute to the bulk signal. We provide a modular atlas that mathematically distinguishes biophysical coronas from these cellular co-isolates, establishing a new framework for interpreting biological origin in plasma EV proteomics.

## Abstract (web summary)

Analyzing 515 clinical proteomes reveals that the plasma extracellular vesicle core is driven by a charge-based corona and physical co-isolation and identifies 8 distinct functional modules, reshaping liquid biopsy biomarker discovery.

## Introduction

Extracellular vesicles (EVs) have emerged as pivotal mediators of intercellular communication and promising sources of liquid biopsy biomarkers. While broadly defined by the International Society for Extracellular Vesicles (ISEV) as non-replicating, lipid-bilayer-delimited particles released by cells^1^, the practical isolation of EVs from complex biofluids yields a heterogeneous mixture of vesicles, vesicle corona and co-isolated non-vesicular (NV) components, such as lipoproteins, protein aggregates, and cellular debris^2^. Consequently, ascribing specific functions or biomarkers to EVs requires a rigorous distinction between vesicular cargo and soluble impurities.

The composition of the core EV proteome remains a subject of intense debate, heavily influenced by the biological source and isolation methodology. In controlled cell culture environments, high-resolution studies have challenged historical assumptions. For instance, Jeppesen et al.^3^ utilized high-resolution density gradients to demonstrate that widely reported EV cargoes, such as DNA, histones, and Argonaute proteins, are largely associated with non-vesicular extracellular matter or amphisomes rather than classical exosomes. Similarly, Kugeratski et al.^4^ showed that the abundance of classical tetraspanins (CD9, CD63, CD81) varies significantly across cell lines, mirroring parental expression rather than a universal vesicle signature, and proposed Syntenin-1 as a more robust ubiquitous marker.

Translating these findings to *human* plasma, however, presents unique challenges due to the vast dynamic range and the presence of blood-specific components. Recent large-scale proteomics efforts have attempted to define plasma-specific signatures. Hoshino et al.^5^ analyzed over 400 *human* samples to define extracellular vesicle and particle (EVP) proteomes, identifying tumor-derived signatures. Addressing the dynamic range, Wu et al.^6^ employed magnetic bead capture (Mag-Net) to uncover signatures distinguishing neurodegenerative conditions. Most recently, Rai et al.^7^ utilized density gradient separation to propose a hallmark panel of 182 proteins capable of distinguishing small EVs from non-EV particles.

Despite these advances, a consensus on the core plasma EV proteome remains elusive. Studies comparing isolation methods, such as Takov et al.^8^, warn that methods yielding higher particle counts (e.g., precipitation or size-exclusion chromatography) often retain significant lipoprotein contamination (e.g., APOB), which can confound functional assays. Critically, it remains unclear to what extent proposed universal plasma EV markers are confounded by the co-isolation of highly abundant blood components, specifically those derived from platelet activation and red blood cell hemolysis.

Collectively, these studies underscore a critical unresolved question distinct from cell culture supernatants: What constitutes the vesicular proteome core of *human* plasma, and which reported markers are merely passengers of co-isolation? Whether defining a core proteome or establishing disease signatures, the assumed EV signature is inextricably linked to the rigor of separation and the presence of dominant components like platelets and erythrocytes.

Therefore, in this manuscript, we aim to resolve this ambiguity through a large-scale meta-analysis of 515 *human* plasma EV proteomes. First, we trace the core isolate to three main mechanistic origins: an electrostatic plasma corona, systematic variations associated with phlebotomy procedures, and biological macromolecular co-isolates. We demonstrate that the reproducible core is overwhelmingly governed by predictable, surface-charge-driven mass-action and, to some extent, ex vivo platelet vesiculation, rather than steady-state vesicle biogenesis. Ultimately, by utilizing a correlation network, we map the highly heterogeneous EV landscape into eight distinct statistical modules reflecting biological origin. This provides a new mathematical framework to interpret biological origin and improve the reproducibility of liquid biopsy studies.

## Results

Previous efforts to define plasma EV signatures were based on the assumption of high EV concentration in the final preparation. Consequently, proteins abundantly identified by LC-MS were often interpreted as defining EV signatures, or at least as EV-associated proteins. The premise in the current study is that plasma EV preparations are complex mixtures comprising multiple molecular components. This conclusion is supported by two key observations: (1) plasma EV preparation is fundamentally different in overall protein composition compared to EVs from cell culture or other biofluids such as urine, pleural effusion, or bronchoalveolar lavage^9^. (2) It is well accepted that MS-based proteomics of plasma samples identifies varying degrees of erythrocytes, platelets, and blood coagulation proteins^10^. It is reasonable to assume that the same protein groups that affect plasma preparations will also plague EV preparations. Additionally, for EVs a non-EV signature for lipoproteins^11^, coagulation^12^, and immune complexes^13^ are expected (Table 1 and Supplementary Data 1).

**Table 1:** Summary of plasma EV anchor proteins

Gene group	Key genes	Reference/Source
RBC lysis	HBB, SLC4A1, CAT, CA1	Pasini et al., Blood ^39^; Bryk & Wiśniewski, J. Prot. Res.^40^
Platelets	ITGA2B, PF4, PPBP	Burkhart et al., Blood ^23^; Geyer et al.,^10^
EVs	CD9, SDCBP, PDCD6IP	Théry et al., JEV (MISEV2018)^2^; Kowal et al., PNAS ^41^
Lipoproteins	APOA1, APOB, LPA	Davidson & Shah, J. Proteomics ^11^
Ig Complexes	JCHAIN, PIGR, IGHM	Kumar et al., eLife ^13^
Coagulation	FGA, FGB, VWF	Anderson & Anderson, Mol. Cell Proteomics ^12^

### A unified proteomics atlas of 515 *Human* plasma EV preparations

**Fig. 1:**
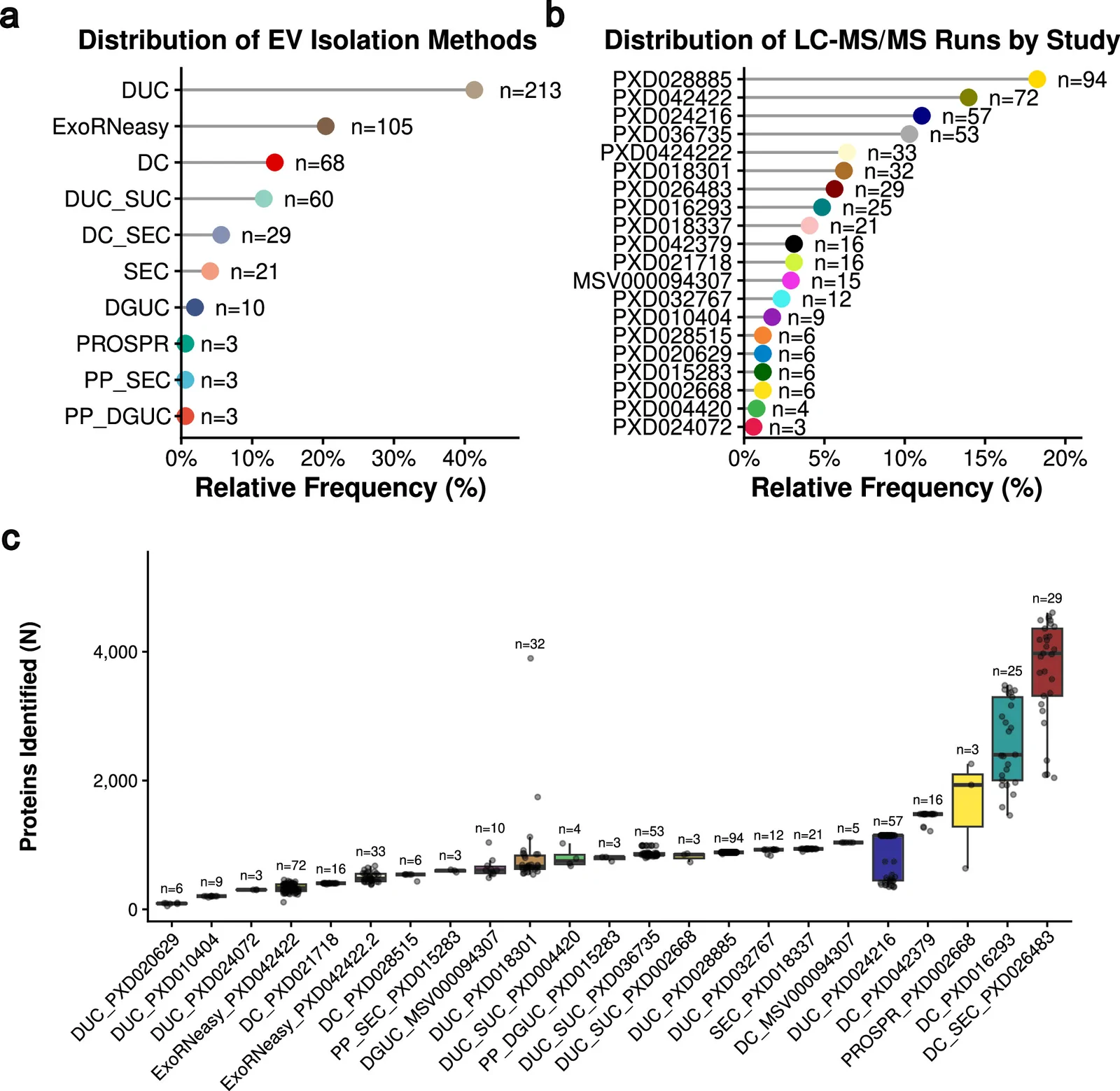
Overview of the 20 plasma EV proteomics studies. **a** Relative frequency of MS runs for each plasma EV isolation methods across 515 LC-MS runs. **b** Number of LC-MS runs per study (ProteomeExchange/PRIDE database entry). **c** Number of proteins identified for each EV isolation method and study. Note few studies used multiple isolation methods. DUC, differential ultracentrifugation; DC, differential centrifugation; SEC, size exclusion chromatography; DGUC, density gradient ultracentrifugation; PP, protein precipitation; PROSPR, PRotein Organic Solvent PRecipitation. ProteomeXchange and MassIVE accession numbers (e.g., “PXD” and “MSV”) denote the specific public datasets analyzed.

To ensure biological specificity and avoid confounding signatures from other sources, our core dataset and all subsequent clustering analyses were restricted exclusively to *human* plasma EV datasets. Data were initially restricted to label free quantification by DDA deposited in ProteomeExchange^14^ and PRIDE^15^. Plasma EV data from ProteomeExchange were extracted from a total of 515 LC-MS files spanning across 20 publications (Fig. 1). In total 10 different EV isolation methods were applied across studies (Fig. 1a). Differential ultracentrifugation (DUC) was the most frequent applied method across the studies and across LC-MS runs (Fig. 1a). The number of LC-MS runs per study ranged from 3 to 94 (Fig. 1b). The number of proteins identified per LC-MS run were for most studies stable with a few displaying variance across samples (Fig. 1c).

The range of proteins identified per study ranged from 142 to 5723, with a mean of 1552 and a median of 1002. The majority of the identified proteins display a stochastic identification frequency. Meaning that these proteins were only identified in a few LC-MS runs (Fig. S1a, b). Fortunately, there is a fairly large subset of 344 proteins (Supplementary Data 2 and Fig. S2) that are consistently identified in more than 50% of all LC-MS runs (Fig. S1a, b insert). We evaluated the consistency of protein identification across independent LC-MS runs and diverse clinical cohorts to define the stability of the circulating EV proteome. The data reveal a proteomics landscape characterized by a highly conserved core proteome amidst a backdrop of extensive biological variation. As expected for plasma, a biofluid that integrates vesicular signals from diverse tissues and pathological states, the majority of the proteome was detected stochastically, resulting in a right-skewed distribution of identification frequencies (Fig. S1a, c). This sparsity reflects the high dynamic range of the plasma proteome and the distinct biological signatures associated with the different pathologies included in this meta-analysis. Rather than representing mere technical noise, this highly variable proteome likely harbors the disease-specific biomarkers required for clinical stratification.

**Fig. 2:**
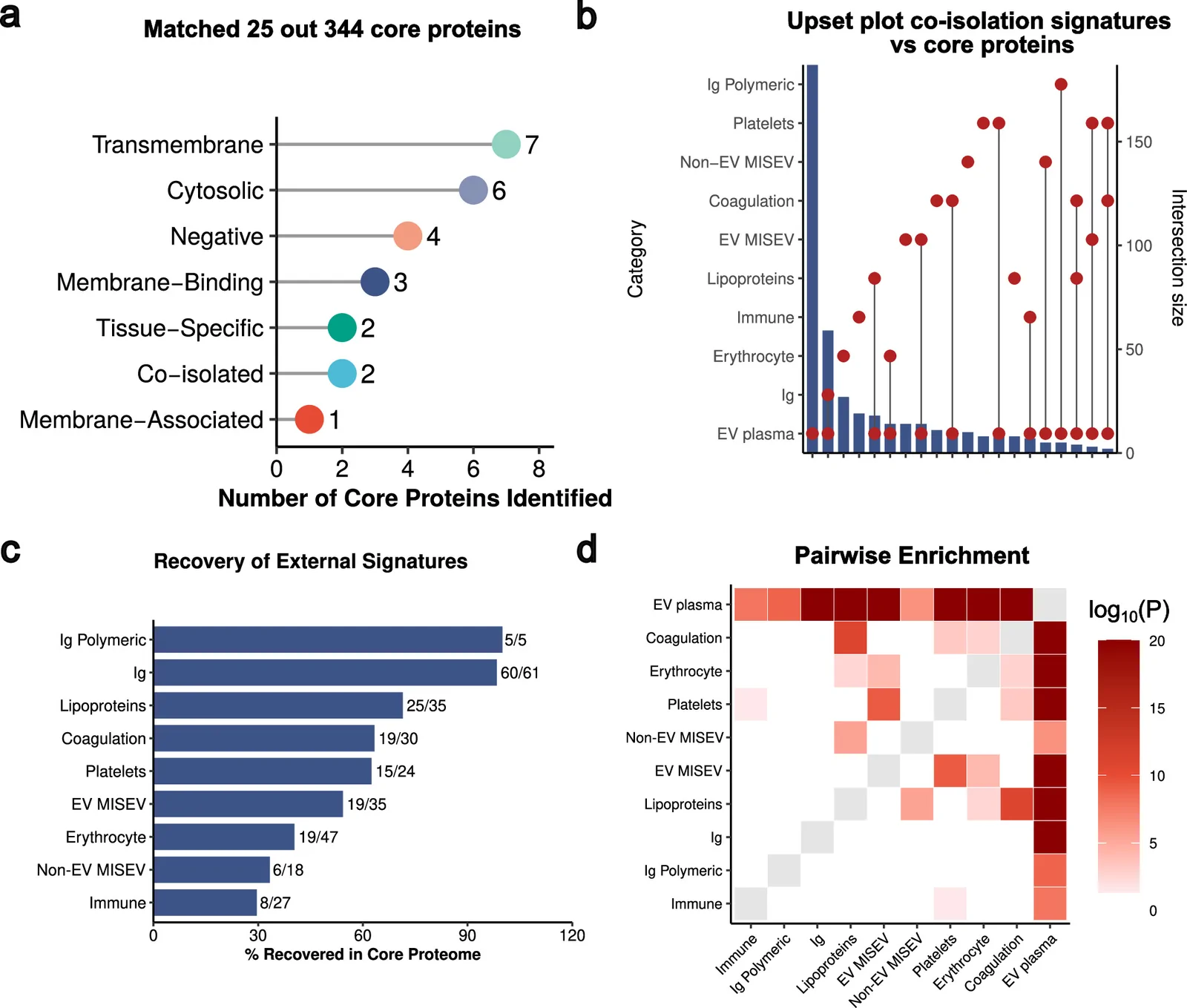
Overlap of core plasma EV proteins with MISEV2018 marker categories and co-isolation signatures. **a** Number of core plasma EVs matching MISEV2018 marker categories. **b** Advanced Upset plot visualizing the intersection between our defined co-isolation signatures and core plasma EV proteins. **c** Recovery plot demonstrating the overlap between our defined co-isolation signatures and core plasma EV proteins. **d** Enrichment heatmap displaying the degree of significant enrichment of co-isolates in plasma EV core proteins, with higher significance represented by red. Self-comparisons (diagonal) are masked in gray to enhance contrast of the off-diagonal enrichment scores. Ig: soluble immunoglobulin.

Remarkably, despite this systemic heterogeneity, we identified a robust core proteome present in the vast majority of samples (Fig. S2). This ubiquitous subset likely represents the fundamental EV structural machinery alongside common co-isolated circulatory cargo, establishing a stable proteomics baseline independent of disease etiology. Notably, 25 of these core proteins are categorized as either EV components or common non-EV co-isolates by the MISEV2018 guidelines^2^. This includes six canonical EV markers: *HSPA8* (Hsc70), *SDCBP* (Syntenin-1), *ADAM10*, *FLOT1* (Flotillin-1), and the tetraspanins *CD9* and *CD81*^16^ (Fig. 2a).

A global heatmap of rank-normalized iBAQ values for the core plasma EV proteome displayed strong clustering of individual LC-MS runs according to their study of origin (Fig. S2). This indicates that technical variables, particularly the EV isolation strategy, significantly influence the quantitative abundance profiles of the core proteome, even among ubiquitously detected proteins.

In summary, a key finding is the consistent detection of 344 proteins across minimum 50% of all LC-MS runs, many of which exhibit similar high protein abundance (Fig. S2). This is encouraging, suggesting a degree of consistency across studies despite variations in pre-analytics, LC-MS sample preparation methods, and label-free LC-MS acquisition, as well as differences in the pathological state of the study subjects.

### Literature review of suspected EV and non-EV markers

Our analysis revealed a significant overlap between the core EV protein panel and established MISEV2018 marker categories. Specifically, 11 core proteins matched MISEV2018 EV membrane proteins and six cytosolic EV membrane proteins. This suggests a degree of alignment with established MISEV2018 EV markers and the plasma EV core proteome (Fig. 2a). Despite this encouraging consistency, several non-EV markers were identified within the core proteome. Additionally, as mentioned above, the premise of this study is that proteins identified in the core proteome of the plasma-isolated EVs are from multiple biological sources. Therefore, literature mining for signatures from different biological sources was performed (Table 1 and Supplementary Data 1). To assess the potential co-isolates within the core plasma EV proteomics data, we analyzed overlaps between our identified core plasma EV proteins and defined co-isolation signatures obtained by literature mining (Fig. 2).

The advanced Upset plot (Fig. 2b) revealed multiple overlaps between potential co-isolate markers and the core plasma EV proteome. Suggesting that a substantial component of non-EV particles was isolated across studies. The recovery plot (Fig. 2c) further supported the presence of co-isolate signatures within the core plasma EV protein panel, demonstrating large overlap with, for example, immunoglobulins, lipoproteins, coagulation, platelets, and erythrocytes. The enrichment heatmap (Fig. 2d) visually confirmed significant enrichment (*P* < 0.05, cumulative hypergeometric distribution) of our defined co-isolate signatures within the core plasma EV protein panel, with the highest levels of enrichment observed for MISEV2018 EV markers, soluble immunoglobulins, lipoproteins, coagulation, platelets, and erythrocytes. In summary, these findings indicate that non-EV components contribute significantly to the core plasma EV proteome.

### Expression level of suspected EV and non-EV proteins

**Fig. 3:**
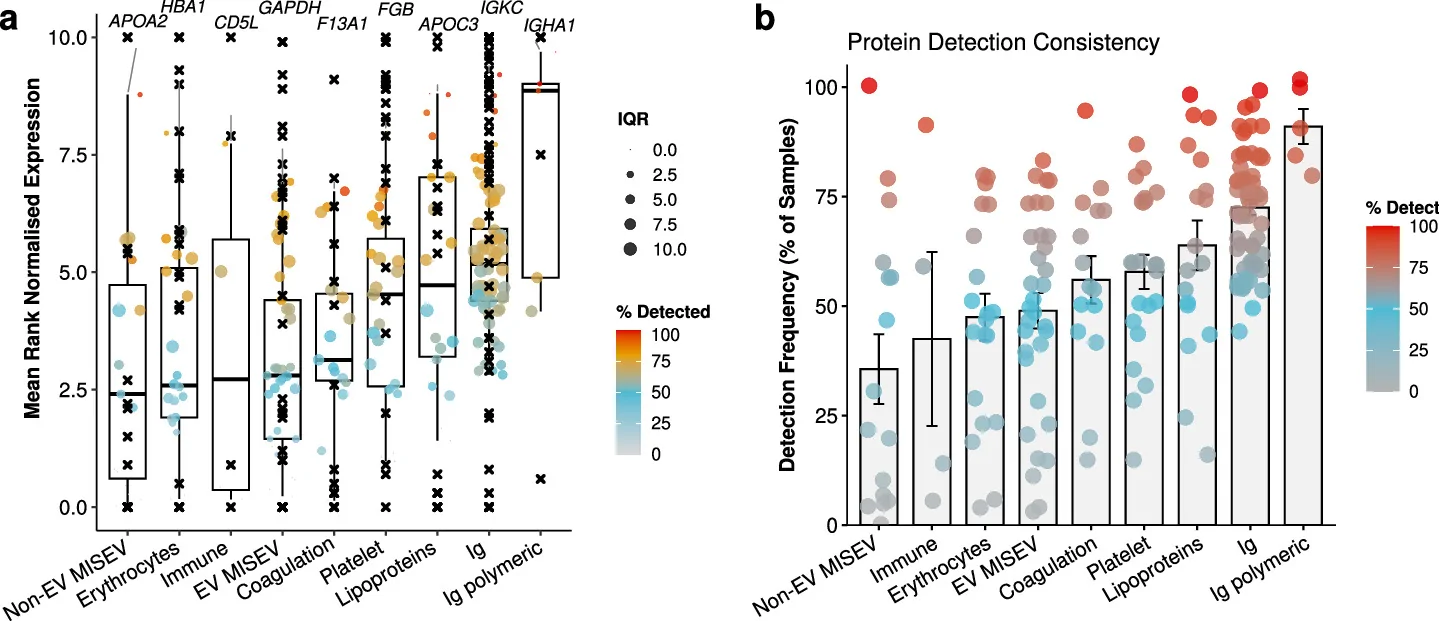
Quantitative abundance and detection consistency of core EV markers in comparison with non-EV markers. **a** Boxplots displaying the mean rank-normalized iBAQ abundance of established EV markers (e.g., CD9, CD81) compared to common non-EV co-isolates (Lipoproteins, Erythrocyte proteins) across the entire dataset. The gene labels above each category indicate the most abundant protein in the core EV plasma proteome for the respective category. The central line represents the median, box limits indicate the 25th and 75th percentiles, and whiskers extend to 1.5× IQR. For the boxplots in a, n represents the number of individual proteins analyzed within each category (Non-EV MISEV *n* = 16, Erythrocytes *n* = 20, Immune *n* = 4, EV MISEV *n* = 33, Coagulation *n* = 15, Platelet *n* = 23, Lipoproteins *n* = 18, Ig *n* = 60, Ig polymeric *n* = 5). The plotted abundance for each protein represents the mean value derived from *n* = 515 biologically independent human plasma EV samples. The crosses indicate abundance values from density gradient isolation of plasma EVs. **b** Detection frequency of each protein category, calculated as the percentage of the total studies in which the marker was identified. In **b**, the bar height represents the mean detection frequency, and error bars indicate the standard error (SE). Note that non-EV proteins are both abundant (**a**) and exhibit high detection consistency compared to core EV markers (**b**). To avoid artificial variance suppression from zero-imputation ties, the Interquartile Range (IQR) of rank-normalized values across samples was calculated strictly across non-zero detection events.

The previous section established that plasma EV proteomes contained proteins with markers originating from suspected EV co-isolates. To further explore the severity of the observed co-isolation, all rank-normalized iBAQ values were compared across co-isolation markers and EV markers (Fig. 3a). It is evident that markers from possible EV co-isolates are highly abundant in comparison to MISEV2018 EV markers. Also, the co-isolation markers extracted from the literature in this study also display a higher abundance than the MISEV2018 non-EV markers. As expected MISEV2018 EV markers displayed a higher abundance than MISEV2018 non-EV markers (Fig. 3a). Overall coagulation, platelets, lipoprotein, soluble immunoglobulin, and polymeric immunoglobulin markers display higher average abundance values than MISEV2018 EV markers (Fig. 3a). Note that the protein categories for this analysis are defined as the core proteins together with proteins that are strongly associated with the category, and these assignments are based on abundance values obtained after isolation of each of the components corresponding to the category (Supplementary Data 3). For example, although abundant in platelets, FGB is synthesized by the liver and endocytosed by megakaryocytes (origin of platelets) from plasma^17^, justifying its classification as a plasma-associated rather than platelet-synthesized protein. Therefore, although FGB is a coagulation factor, its sequestration in alpha-granules means it is frequently associated with platelet contamination in EV preparations. Many of the core and strongly associated markers are shared among multiple signature categories, which complicate marker definition. Figure 3a was created from a strict list of consensus markers (Supplementary Data 3). However, similar results of abundant non-EV protein signatures were obtained for a less strict definition of signatures (Fig. S3a and Supplementary Data 1). High average expression of non-EV proteins might be caused by a few studies displaying abnormally high co-isolation. To exclude this possibility, the detectability across studies (Fig. 3b) and LC-MSMS runs (Figure S3b) were calculated. Around half of the proteins in the signatures were identified in more than 75% of all studies and almost all in more than 50% of the studies (Fig. 3b).

To rigorously validate these findings and address whether the dominant abundance of non-EV proteins was dependent on sparsely detected artifacts, a sensitivity analysis was performed applying progressively strict detection frequency thresholds (30%, 70%, and 90% of studies; Supplementary Fig. S4). Strikingly, as the stringency increased, the disparity between EV and non-EV markers became even more pronounced. At the highly stringent 90% detection threshold, canonical MISEV2018 EV markers failed to meet the criteria and were eliminated from the analysis entirely (Supplementary Fig. S4c). In contrast, massive plasma co-isolates, specifically lipoproteins, soluble immunoglobulins, and polymeric immunoglobulins, remained consistently detected at high abundance. This demonstrates that the dominant signal in plasma EV preparations is mainly high-abundance non-EV networks.

Overall, non-vesicular proteins are highly abundant in plasma EV preparations, exhibiting near-ubiquitous detection frequencies across independent LC-MS studies. Within this matrix of co-isolates, circulating immunoglobulins and lipoproteins consistently account for the highest quantitative mass in the EV fractions.

### Quantification of relative co-isolate burden using internal EV markers as reference

To complement the rank-based abundance analysis and rigorously control for fluctuations in overall EV yield across the 515 LC MS runs, we evaluated the purity of EV preparations using an internal biological control. We calculated the “Relative Co-isolate Module Burden”, which normalizes the log-transformed quantitative signal of specific plasma co-isolate modules directly against the corresponding signal of canonical EV structural markers (established by MISEV guidelines) detected within the exact same sample. In this metric, a score greater than zero indicates that the cumulative signal of the co-isolate module exceeds the signal of the core EV structural proteins.

**Fig. 4:**
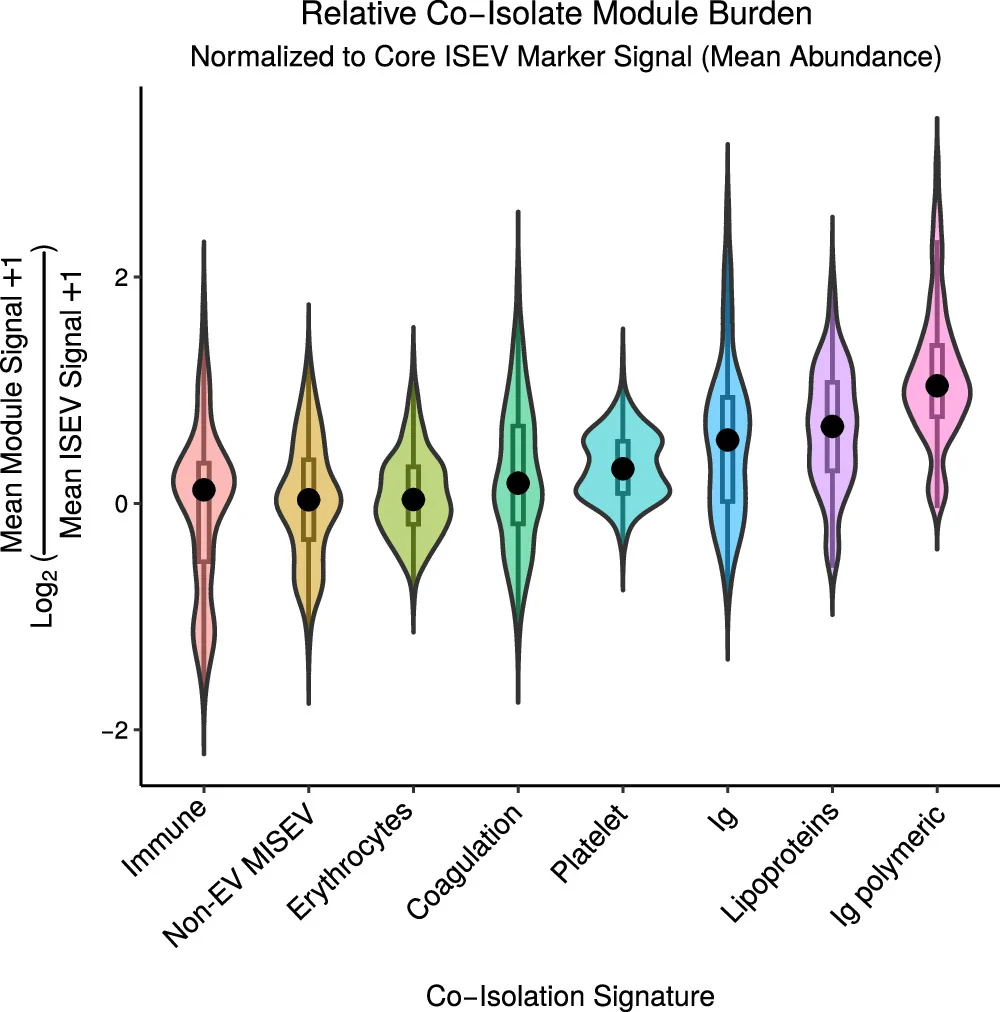
Global assessment of relative co-isolate module burden across 515 DDA plasma EV preparations. Box plots display the relative abundance of distinct plasma co-isolation modules normalized against an internal vesicular control (log_2_ ratio; see Methods).

Consistent with the rank-normalized data, the global DDA meta-analysis revealed a pervasive and dominant co-isolate burden across plasma EV preparations (Fig. 4). Notably, modules representing polymeric immunoglobulins (Ig polymeric) and lipoproteins consistently exhibited mean burden scores above zero. This indicates that in standard plasma EV preparations, the quantitative mass spectrometry signal is fundamentally overwhelmed by these co-isolated plasma networks rather than vesicular cargo.

We next investigated whether specific physical EV isolation methodologies could partially remove EV co-isolates. Stratification of the DDA datasets by isolation technique (e.g., ultracentrifugation, size exclusion chromatography, precipitation) demonstrated that a substantial co-isolate burden remains a ubiquitous phenomenon (Fig. S5). While slight variations existed between methods, no single physical isolation technique successfully inverted the ratio to consistently yield preparations where EV marker signals dominated the high-abundance plasma modules such as lipoproteins.

To orthogonally validate these findings against alternative quantitative mass spectrometry modalities, we applied the relative burden metric to recent Data-Independent Acquisition (DIA) datasets from Suresh et al.^18^ (Fig. S6) and Wu et al.^6^ (Fig. S7). Importantly, addressing the premise that DIA’s superior quantitative accuracy might minimize the appearance of these co-isolates, the DIA data firmly corroborated the DDA meta-analysis, demonstrating that the co-isolation burden is actually just as prominent, if not more so, across these methodologies (Fig. S6). Furthermore, the relative burden metric proved highly sensitive to the biological context of the starting material. In the Wu et al.^6^ dataset, the platelet module exhibited the highest relative burden of all evaluated signatures (Fig. S7). This exceptionally high platelet burden is biologically and methodologically consistent, as Wu et al.^6^ specifically utilized platelet-enriched plasma for their EV isolations. This specific alignment demonstrates that the relative module burden accurately and robustly captures the dominant background elements inherent to the initial biofluid state. While Wu et al.^6^ demonstrated the overall best (lowest) burden for the remaining plasma modules, indicated by scores below zero, this exceptional result precludes direct comparison with the other datasets. The vast majority of studies followed ISEV guidelines utilizing centrifugation protocols to obtain platelet-depleted plasma, whereas the Wu et al.^6^ study applied a centrifugation method yielding platelet-enriched plasma, which fundamentally skews the baseline. Rather than precluding a fair purity comparison, this powerfully validates the sensitivity of our metric to the initial source material. Ultimately, these cross-platform findings emphasize that the co-isolate burden is a physical reality of the samples rather than an artifact of DDA mass spectrometry, further reinforcing the need for computational deconvolution to investigate the EV core.

### Concentration-driven bias and the core plasma EV proteome

**Fig. 5:**
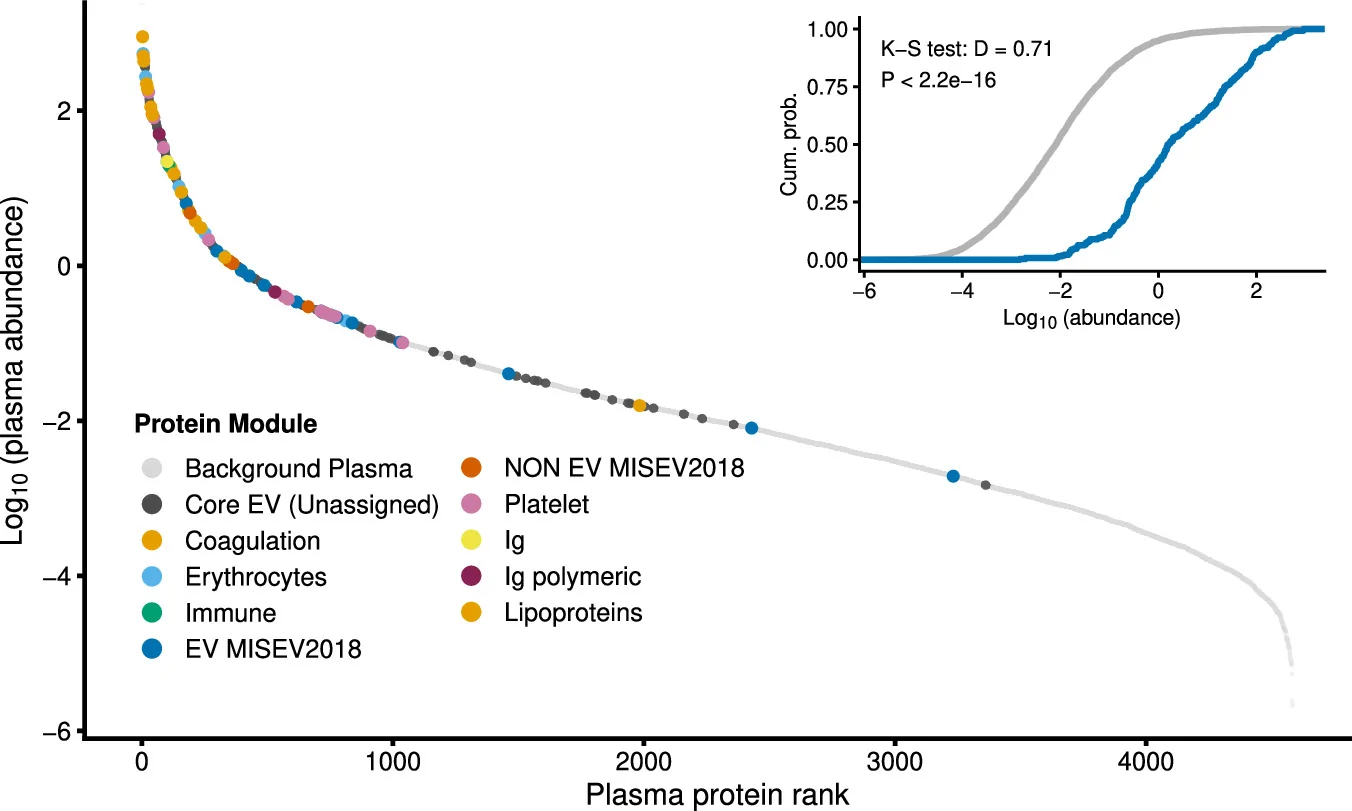
The core plasma EV proteome overwhelmingly samples the most abundant proteins in human plasma. A rank-abundance curve of the comprehensive physiological human plasma proteome derived from the PeptideAtlas database. Gray background points represent the entire detectable plasma proteome sorted by descending Log_10_ abundance. Colored points highlight the proteins that comprise the defined core plasma EV proteome, stratified by their assigned co-isolation module signatures. Inset: Empirical cumulative distribution function (ECDF) comparing the Log_10_ abundance of the background plasma proteome (gray line) against the core plasma EV proteome (blue line).

To contextualize these prevalent co-isolates within the native physiological state of the biofluid, we mapped the empirically defined core plasma EV proteome directly onto the comprehensive physiological *human* plasma proteome (derived from the PeptideAtlas database)^19^. Visualizing this relationship via a rank-abundance curve reveals a profound mass-action bias (Fig. 5). Rather than representing a distinct, low-abundance vesicular signature, the proteins most frequently and consistently reported as core to plasma EVs heavily cluster at the extreme upper stratum of physiological plasma abundance.

This concentration-driven sampling bias is robustly confirmed by comparing the cumulative distribution functions (CDF) of the two populations. As shown in the inset of Fig. 5, the abundance distribution of the core plasma EV proteome is massively shifted toward higher concentrations relative to the background plasma proteome (two-sided Kolmogorov-Smirnov test: *D* = 0.71, *P* < 2.2 × 10^−16^). Crucially, when stratifying the core EV proteins by their co-isolation signatures on the main curve, canonical EV markers (EV MISEV2018) distribute further down the physiological concentration gradient. Conversely, the absolute peak of the curve is almost entirely dominated by highly concentrated plasma networks including lipoproteins, immunoglobulins, and coagulation factors. This profound dependence on background fluid concentration strongly suggests that the dominant proteomic profile of plasma EV preparations is driven by mass-action protein-nanoparticle interactions, effectively forming a biomolecular corona during isolation, rather than representing natively sorted intra-vesicular cargo.

### The core EV proteome mirrors surface-charge-driven nanoparticle coronas

Having established that standard EV isolation is overwhelmed by a concentration-driven bias, we next investigated the biophysical mechanism driving this co-isolation. By calculating the abundance-weighted isoelectric point (pI) of the core EV proteome, we identified an abundance-weighted pI of 6.44, falling within the electrostatic continuum of protein coronas formed on engineered synthetic nanoparticles (Blume et al.^20^, ranging from 5.6 to 6.9, Supplementary Data 4).

To evaluate stoichiometric similarity, we utilized a custom Coverage-Weighted Spearman’s rank correlation. The synthetic nanoparticle that most accurately mirrored the EV core abundance profile (Sample X2925, Coverage-Weighted *ρ* = 0.48, raw *ρ* = 0.55, *p* = 1.57 × 10^−25^) exhibited a weighted pI of 5.8. While the abundance profiles correlate strongly (Figure S8, Supplementary Data 5), the slight shift in the overall EV pI toward neutrality (6.44) reflects the inherent structural complexity of a biological lipid bilayer compared to a uniform synthetic polymer. Nonetheless, this biophysical convergence suggests that the bulk of the core EV proteome is governed by predictable, surface-charge-driven mass-action.

### The reproducible core plasma EV isolate: decomposition into three mechanistic origins

To definitively characterize the cellular origin of the remaining core plasma EV proteins, we cross-referenced our dataset against the proteomic signatures of isolated platelet-derived EVs (pEVs) generated via distinct physiological agonists^21^. Utilizing our custom coverage-weighted Spearman’s rank correlation, we evaluated the stoichiometric similarity between the plasma EV core and pEVs stimulated by ADP, collagen, thrombin, and a thrombin/collagen dual-stimulation (Supplementary Data 6).

The plasma EV core exhibited the strongest quantitative alignment with pEVs stimulated by thrombin, an agonist essential for potent and prolonged platelet activation typically found within the core of a thrombus. This specific correlation suggests that the vesicular component of widely accepted plasma EV preparations is not predominantly comprised of steady-state, natively circulating EVs. Rather, it is also driven by ex vivo platelet vesiculation triggered by shear stress, contact activation, and localized thrombin generation during standard phlebotomy and blood processing.

To quantitatively decompose the EV core into its distinct origins, we mapped the rank-abundance distribution of the core proteome against both its biophysical (corona) and cellular (platelet) drivers. Crucially, while this platelet-derived signature defines the vesicular compartment, the overall quantitative correlation between the plasma EV core and these optimal platelet preparations was lower in magnitude than the correlation observed with the synthetic nanoparticle corona. We initially evaluated the intersection between the single optimal synthetic corona (Blume Sample X2925) and the optimal platelet activation state (Thrombin pEVs) (Fig. S9 and Supplementary Data 7). While this intersection accounted for a substantial portion of the proteomic mass, we hypothesized that a single synthetic polymer surface could not perfectly capture the entire biophysical capture spectrum of a complex lipid bilayer.

**Fig. 6:**
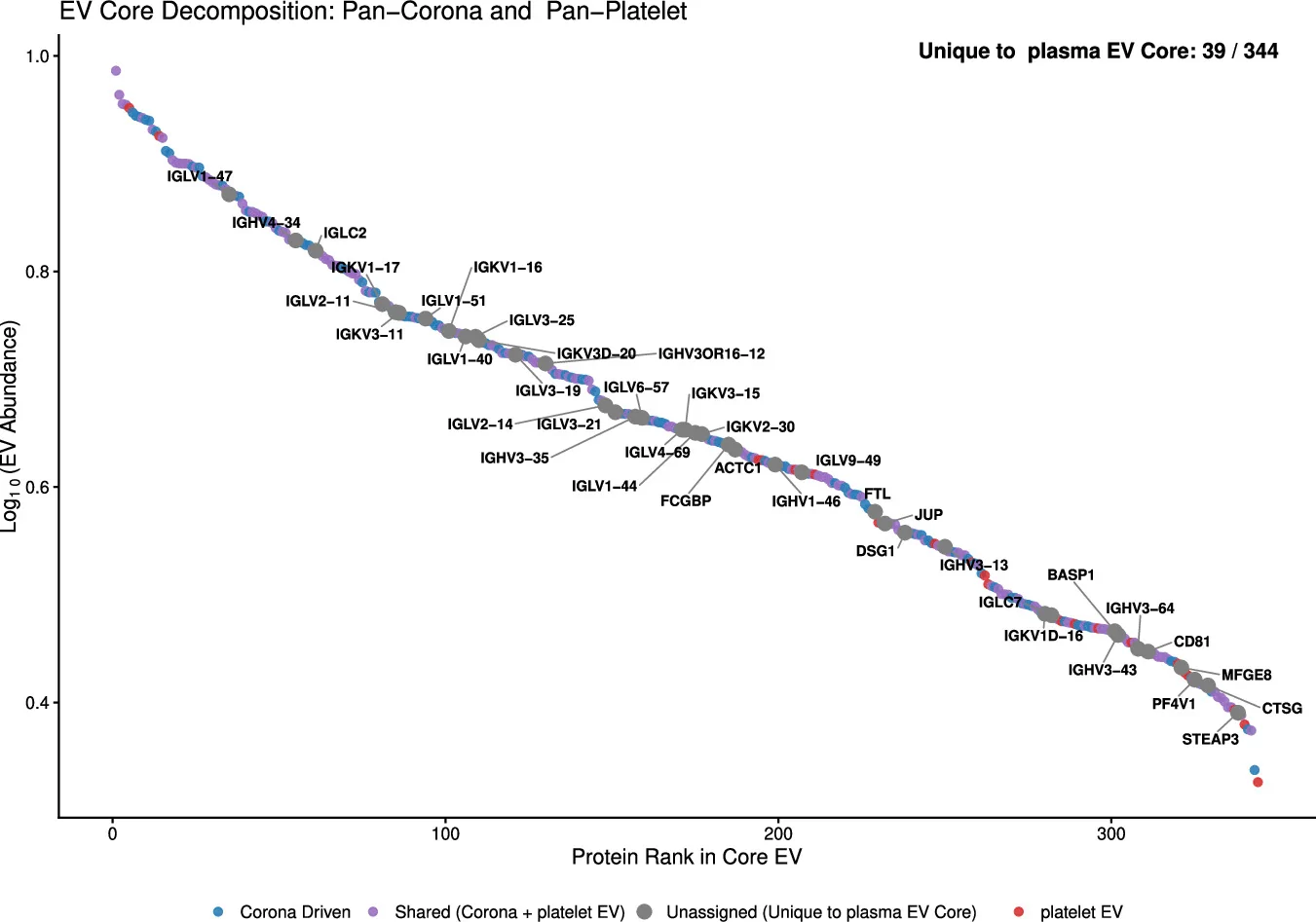
Comprehensive stoichiometric decomposition of the core plasma EV proteome. Rank-abundance distribution of the core plasma EV isolate (344), partitioned by its quantitative intersection with a comprehensive pan-corona reference (comprising the maximum abundance across all 11 synthetic nanoparticle chemistries from Blume et al.) and a pan-platelet cellular reference (comprising the maximum abundance across all agonist-induced platelet EV states). Protein origins are categorized as follows: Corona Driven (blue) represents mass-action proteins captured exclusively by synthetic nanoparticle surfaces; Vesicle Driven (red) represents functional cargo found exclusively in ex vivo platelet-derived EVs; and Shared (purple) indicates proteins quantified in both biophysical and cellular reference datasets. The Unassigned fraction (gray, 39) comprises the residual proteins mapping to neither the electrostatic plasma corona nor ex vivo platelet vesiculation. Enlarged gray points are annotated with their respective gene names, revealing a highly specific biological corona module overwhelmingly dominated by circulating immunoglobulins (e.g., JCHAIN, IGHV, IGKV, IGLV) and apolipoproteins (e.g., APOC2, APOC4) whose co-isolation strictly depends on the structural presence of a native lipid bilayer.

To determine if any portion of the core EV proteome exists independently of generalized surface-charge-driven mass-action or general platelet degranulation, we expanded our analysis to a comprehensive pan-corona (the maximal abundance across all 11 synthetic nanoparticle chemistries) and pan-platelet (the maximal abundance across all agonist-induced pEV states) intersection (Fig. 6 and Supplementary Data 8). Strikingly, this comprehensive pan-intersection captured the vast majority of the quantitative mass of the plasma core EV isolate. Out of 344 core proteins, only 39 remained unassigned to either a synthetic corona or a platelet EV origin (Fig. 6). By designating these residual proteins as biological macromolecular co-isolates, we successfully map the entirety of the reproducible plasma EV core back to three main mechanistic origins.

Crucially, these 39 remaining unique proteins are not canonical intra-vesicular markers. A systematic review of their identities reveals an overwhelming dominance of circulating immunoglobulins (e.g., IGHV, IGKV, IGLV, JCHAIN), apolipoproteins (APOC2-APOC4), and acute-phase reactants (SAA2-SAA4). The inability of 11 distinct synthetic polymer surfaces to capture this specific subset of proteins highlights a critical biophysical divergence. While the bulk of the plasma EV isolate is governed by basic electrostatic corona formation (captured by the beads) and ex vivo platelet artifacts (captured by the pEVs), this final biological corona module relies on structural interactions that strictly require a native lipid bilayer, such as insertion into lipid rafts, surface glycan binding, indirect protein interaction, or Fc-receptor-mediated associations.

### The reproducible core plasma EV isolate: co-isolation of functional modules

**Fig. 7:**
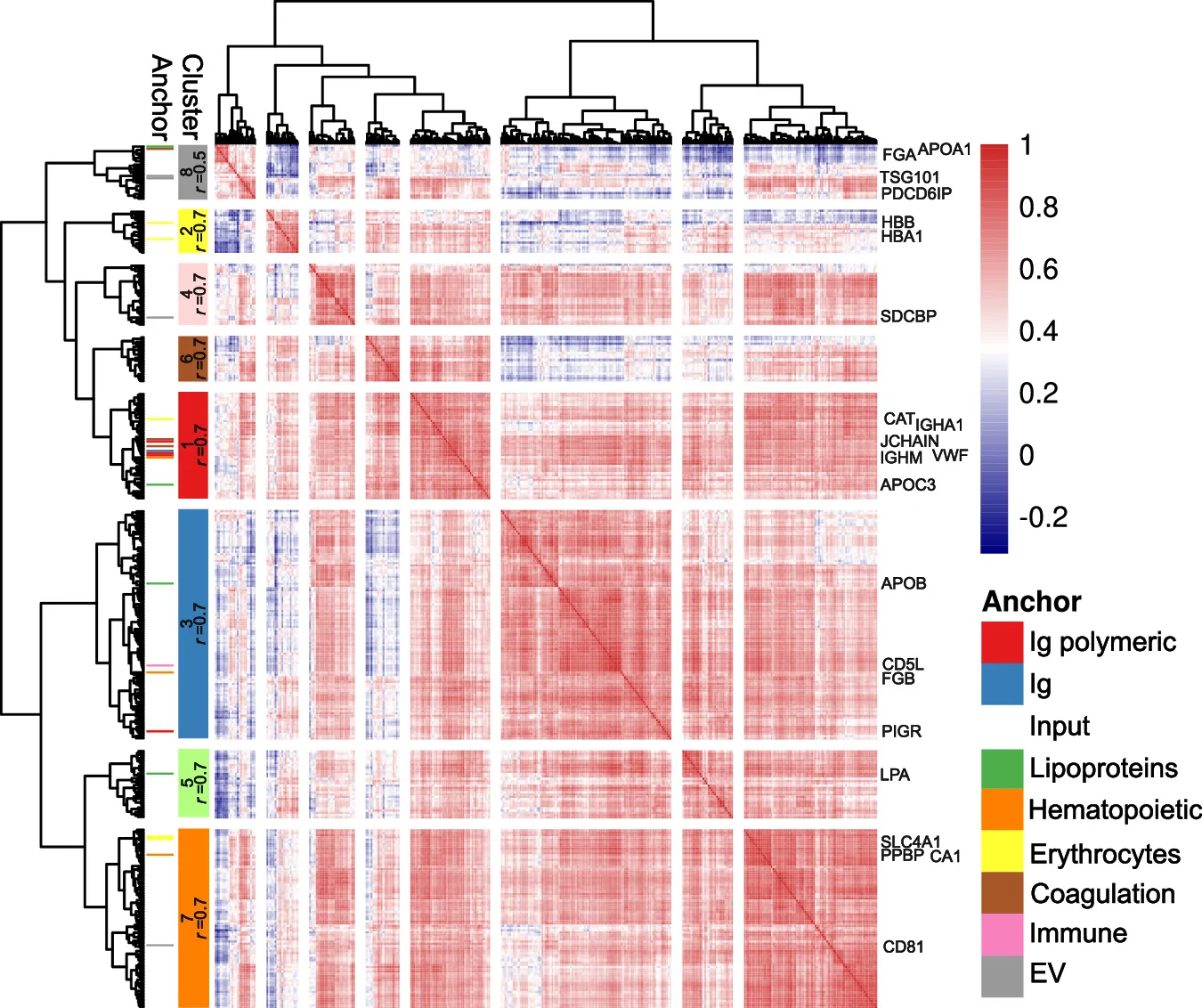
Deconvolution of the plasma EV core proteome and anchor proteins into eight distinct biological modules. Pairwise correlation analysis of the core proteome reveals eight co-isolating protein clusters. Biological annotation was performed using the anchor proteins defined in Table 1. The side annotation bar indicates the specific biological category of individual proteins. Cluster-level color coding represents the predominant biological category identified within that module (determined by the most frequent anchor protein assignment); clusters lacking a dominant category are assigned a distinct color. Cluster labels indicate the Cluster ID and the mean correlation coefficient (*r*) of the module. Heatmap intensity represents the pairwise Pearson correlation between proteins.

To deconvolute the biological architecture of the plasma EV isolate, we restricted our analysis to the core proteome, defined as the 344 proteins consistently quantified in 50% of the 515 samples. This high-frequency dataset represents the stable, reproducible proteome background of clinical plasma EV preparations. We applied unsupervised hierarchical clustering to the pairwise correlation matrix derived from the rank-normalized protein abundances to identify co-varying functional units (Fig. 7) and subsequently computationally validated these findings using a targeted candidate marker panel (Fig. S10).

### The core proteome: dominance of soluble and hematopoietic backgrounds

Clustering analysis of the unbiased core proteome (Fig. 7 and Supplementary Data 9) revealed that the reproducible EV isolate is structurally dominated by non-vesicular plasma components and blood cell membrane fragments rather than generic tissue-derived vesicles.

The majority of the proteome segregated into high-abundance soluble modules driven by macromolecular size or surface adsorption. Cluster 1, was enriched in polymeric immunoglobulins (*JCHAIN, IGHA1, IGHM*) and large glycoproteins (*VWF*), representing massive molecular complexes that likely co-pellet due to their hydrodynamic radius (Supplementary Data 9 (*r* = 0.72)). Similarly, Cluster 3, captured the Immune Effector Complex, defined by the tight co-regulation of the C1 complement complex (*C1QB, C1QC*) and fibrinogen (*FGB*) (Supplementary Data 9 (*r* = 0.70)). This cluster likely reflects the electrostatic plasma corona or immune complexes adsorbed to the vesicle surface.

Crucially, the primary cell-derived signature in the core proteome (Cluster 7, Supplementary Data 9, *r* = *0.74*) was a chimeric hematopoietic-derived EV network, driven by the co-isolation of platelet (*ITGA2B, GP1BB, CD36*) and erythrocyte (*SLC4A1*) membrane proteins. Definitive EV markers often cited in the literature, including CD9, CD81, and ADAM10, clustered within this hematopoietic module. This strongly suggests that the reproducible tetraspanin signal in plasma is driven by the bulk recovery of blood cell microvesicles rather than a generic EV population.

Finally, the core analysis identified a specific endosomal/ESCRT signature in Cluster 8, anchored by the canonical markers TSG101 and ALIX (*PDCD6IP*) (Supplementary Data 9 (*r* = 0.54)). Notably, in this unbiased analysis, Apolipoprotein A-I (APOA1), the primary scaffold of HDL, also co-clustered with these markers. This indicates that while the ESCRT machinery forms a detectable variance component in the core proteome, its elution profile partially overlaps with High-Density Lipoproteins.

### Structural deconvolution of consensus EV signatures

To benchmark our findings against the current consensus in the field, we applied our clustering framework to the extensive panel of EV features reported by Rai et al.^7^. This dataset serves as a representative inventory of proteins frequently cited across the literature as plasma EV biomarkers. Our objective was to determine whether these widely reported features constitute a homogeneous vesicular signature or a composite of the broader plasma milieu. Unsupervised clustering of this representative feature set revealed that the canonical plasma EV signature is not a singular biological entity, but rather a composite of at least eight statistically distinct variance components (Fig. S10 and Supplementary Data 10). This analysis effectively disentangles the vesicular signals from the reproducible co-isolates that frequently confound plasma proteomics: (1) The hematopoietic-derived EV network (Cluster 5 & 7). Consistent with our Core analysis, the strongest variance component in this consensus dataset (Cluster 5, *r* = 0.77) defined a blood cell membrane signature. This module confirmed that canonical tetraspanins (CD9, CD81) and ADAM10, central pillars of EV definitions in the field, cluster inextricably with definitive platelet (ITGA2B) and RBC (SLC4A1) surface markers. This stratification clarifies a pervasive ambiguity in the field: the EV proteome signal in bulk plasma is primarily driven by the burden of hematopoietic-derived EVs than generic tissue-derived EV secretion. (2) Resolution of the endosomal vs. lipoprotein signal. A major strength of this structural analysis was the resolution of the ESCRT machinery from lipoprotein signals. While these two classes are often conflated in literature-derived lists, our clustering separated the endosomal markers TSG101 and PDCD6IP (ALIX) (Cluster 8, *r* = 0.77) from the soluble/micellar module containing Apolipoprotein A-I (Cluster 6). This demonstrates that while both vesicle types and HDL are present in consensus feature sets, they behave as independent biological variables that must be interpreted separately. (3) The misassignment of the soluble host response. Crucially, the clustering analysis revealed that a significant proportion of features broadly accepted as EV markers segregate into soluble plasma modules. Cluster 3 identified the Immune Effector Complex (C1q, Fibrinogen), while Cluster 1 captured soluble immunoglobulin complexes. The distinct segregation of these clusters from the membrane modules (Clusters 5 & 8) suggests that their frequent reporting in EV studies reflects their reproducible presence as part of the protein corona or soluble background, rather than vesicular encapsulation. In summary, by stratifying this representative dataset, we demonstrate that many proteins currently ascribed to the plasma EV proteome are actually markers of the soluble host response (immune complexes, coagulation) and hematopoietic turnover. While these non-vesicular co-isolates may possess diagnostic utility as surrogates for systemic disease, distinguishing them from vesicular cargo is essential for accurately defining biological origin.

## Discussion

### Beyond purity: a modular anatomy of the plasma EV isolate

The pursuit of a highly enriched plasma EV signature has long been hindered by the complexity of the blood proteome, where vesicular signals are masked by soluble proteins and lipoprotein particles. The presence of non-EV proteins in physical EV isolates is now widely recognized in the field, supported by recent comprehensive meta-analyses by Vallejo et al.^14^. However, our 515-sample deconvolution builds upon this established consensus by demonstrating that these non-vesicular proteins do not appear as stochastic background noise; rather, they resolve into highly structured, reproducible biological networks. By integrating plasma abundance mapping, synthetic nanoparticle coronas, and agonist-induced platelet profiles, we first defined the core EV isolate through three fundamental mechanistic origins: an electrostatic plasma corona, pan-platelet, and biological macromolecular co-isolates. Importantly, recognizing this corona acknowledges that many co-isolates are not merely freely soluble noise, but rather represent biologically relevant proteins tethered to EVs through indirect interactions, becoming secondarily recruited to the vesicular surface during circulation. Crucially, by subsequently applying co-variance network analysis across 515 clinical proteomes, we demonstrate how these underlying physical forces manifest in the patient population as eight distinct statistical modules. By resolving this architecture, we shift the analytical paradigm from simple inclusion lists to biophysical stratification. We show that high-abundance proteins frequently cited as EV markers do not behave as a homogeneous group, rather, they segregate into mutually exclusive components, ranging from the hepatic secretome^22^ and immune effector complexes to discrete hematopoietic-derived EV networks and endosomal populations. This modular atlas explains why reproducibility in plasma proteomics remains elusive: independent studies routinely conflate distinct biological variables, such as liver function, immune activation, and platelet shedding, under the singular label of EVs. This conclusion strongly validates and scales the foundational observations of Rai et al^7^. While their work elegantly demonstrated that classical EV features often co-elute with major non-vesicular plasma components, our meta-analysis provides the mathematical architecture mapping exactly how these specific non-vesicular components consistently form distinct, reproducible physical modules across hundreds of clinical samples and disparate isolation techniques. Notably, this architectural stability is further corroborated by our data-independent acquisition (DIA) analyses. The DIA data proved to be robust across various purification methods and accurately reflected the diverse nature of the starting materials, such as those detailed in the Wu et al. study (Supplementary Fig. S6).

### The divergent biogenesis of canonical markers: shedding vs. sorting

A critical finding of this study is the definitive schism between the two primary modes of vesicle biogenesis in plasma. Current MISEV guidelines often group tetraspanins (CD9/CD81) and ESCRT machinery (TSG101/ALIX) as interchangeable markers of EV presence. Our data fundamentally overturn this assumption for plasma-derived samples.

We observed a significant clustering of EV markers CD9, CD81, and ADAM10 with the platelet (ITGA2B, GP1BB) and erythrocyte (SLC4A1) surface proteomes (Cluster 5, Supplementary Data 10 and Cluster 7, Supplementary Data 9). Rather than representing a uniform population of generic tissue EVs, this co-variance indicates a strongly associated, composite hematopoietic-derived EV network. The specific lineage contributions within this network are corroborated by orthogonal datasets. Cross-referencing with deep-proteomic profiles of agonist-induced platelet EVs^21^ and platelets^23^ confirms that CD9 and ADAM10 are highly abundant in, and thus likely driven by, bulk platelet vesiculation, aligning with recent flow cytometry evidence that platelet EVs constitute the dominant vesicular fraction in blood^24,25^. In contrast, CD81 is notably absent from these platelet secretomes. Instead, CD81 is a well-established surface marker for circulating lymphocytes, functioning as a core receptor component on B and T cells^26^. Therefore, the shared mathematical variance of these markers likely reflects the biophysical co-isolation of EVs from distinct blood cell lineages (platelets and lymphocytes). Crucially, this proteomic conclusion is strikingly concordant with recent transcriptomic evaluations; for example, Li *et al*.^27^ analyzed long RNA profiles and independently concluded that the bulk plasma EV signal is overwhelmingly dominated by hematopoietic ectosomes rather than generic tissue-derived EVs. The convergence of these multi-omic datasets reinforces our caution that elevations in CD9 or ADAM10 in liquid biopsies primarily reflect local hematopoietic activation or systemic turnover, rather than disease burden.

In contrast, the canonical ESCRT machinery (TSG101, ALIX) formed a statistically distinct module (Cluster 8, Supplementary Data 9 and 10) that did not co-vary with the primary tetraspanin networks. While the overall MS intensity of this ESCRT module was lower, we note the inherent limitations of abundance-based inference in proteomics; this lower signal may reflect the low per-vesicle stoichiometry of ESCRT components rather than a low absolute number of vesicles. Nonetheless, the statistical separation of these markers suggests that ESCRT-associated vesicles possess an independent regulatory or biogenesis network distinct from the dominant hematopoietic-derived pool. This mirrors findings from high-resolution iodixanol gradient studies^3^, which demonstrated the physical separation of tetraspanin-rich and ESCRT-rich vesicles, confirming this dichotomy is maintained across complex clinical plasma cohorts.

### The functional matrix: redefining background noise as structured co-isolation

As established by our cross-referencing with the *Human* Plasma PeptideAtlas and synthetic nanoparticle arrays, the EV core is overwhelmingly dominated by a surface-charge-driven electrostatic corona. While the integration of corona composition data based on the Blume et al.^20^ study provides valuable mechanistic insights into this phenomenon, we acknowledge that reducing the EV binding properties of corona proteins solely to their isoelectric point (pI) is a simplification. In biological fluids, these nanoparticle-protein interactions are inherently multifactorial and are likely governed by a complex interplay of surface charge, steric hindrance, localized hydrophobicity, and specific lipid bilayer composition. Our co-variance network analysis further reveals that these high-abundance soluble proteins do not fluctuate randomly across the clinical population; rather, they segregate into highly reproducible statistical modules. Rather than dismissing these co-isolates as random noise, we mathematically characterize them as the functional biophysical matrix of the plasma EV isolate.

Specifically, Cluster 3, Supplementary Data 9 identified the immune effector complex, where the C1q complement machinery and fibrinogen tightly covary with immunoglobulins. This likely represents the protein corona^28^ or circulating immune complexes (CICs) that possess biophysical properties (size/density) overlapping with EVs. Similarly, Cluster 5, Supplementary Data 9 captured the hepatic secretome (CRP, SAA, Hemopexin), representing the soluble acute-phase response. Recognizing these modules is vital for biomarker discovery: a proteomic signature in a cancer patient may reflect the systemic hepatic response (Cluster 5) or systemic coagulation (Cluster 3/6, Supplementary Data 9) rather than tumor-derived vesicular cargo.

### Deconvoluting cell lysis from active secretion

Quality control in plasma proteomics has historically relied on single markers like hemoglobin. Our modular analysis provides a higher-resolution metric by distinguishing cytosolic lysis (Cluster 4, Supplementary Data 10: PKM, HBB, HBA1) from active secretion (Cluster 7, Supplementary Data 10: RAB27B, VASP).

The segregation of these clusters is biologically significant. It demonstrates that the release of cytosolic enzymes (lysis) and the shedding of membrane vesicles (secretion) are independent variance components in clinical samples. Crucially, the statistical independence of platelet-derived EVs (Cluster 5, Supplementary Data 10) from lysis markers (Cluster 4, Supplementary Data 10) confirms that the vesicular signal derives from active cellular shedding rather than passive membrane fragmentation. Conversely, the co-clustering of mitochondrial proteins (ATP5F1B) with the lysis module serves as a specific discriminator for leukocyte or platelet disintegration, distinguishing these events from simple hemolysis, as mature erythrocytes are entirely devoid of mitochondria^29–31^. A fundamental limitation of any retrospective meta-analysis is the inability to fully control for pre-analytical variables. The historical datasets integrated here exhibit pervasive gaps in metadata reporting regarding plasma preparation, freeze-thaw cycles, and biobanking storage duration. While we cannot computationally model these missing variables, dedicated studies on EV stability suggest that while storage conditions impact absolute yield and functional activity, the core structural proteome and dominant co-isolates remain relatively stable^1,32^. Furthermore, the robust emergence of our functional modules indicates that the variance driven by physical isolation methodology supersedes the underlying pre-analytical noise. Nonetheless, this unmeasured heterogeneity underscores the critical importance of standardized MISEV reporting guidelines for future multi-omic studies. Furthermore, a specific technical limitation of our deconvolution approach is the reliance on a >50% detection threshold across the clinical cohorts to construct the statistical networks. While this stringency is mathematically necessary to resolve the co-variance architecture of the core plasma isolate, it inherently acts as a high-pass filter. Consequently, the modules defined here fundamentally map the dominant, ubiquitous biophysical background of plasma (i.e., the bulk hematopoietic EVs and the co-fractionating functional matrix). We explicitly recognize that this approach actively filters out and hinders the detection of rare, tissue-specific EV biomarkers, which may only be present in a small fraction of disease-specific clinical samples. Therefore, while this atlas provides a necessary biophysical map of the plasma background, targeted or highly enriched analytical approaches remain essential for the detection of low-abundance, tissue-derived vesicular cargo.

### Toward a component-based framework

**Fig. 8:**
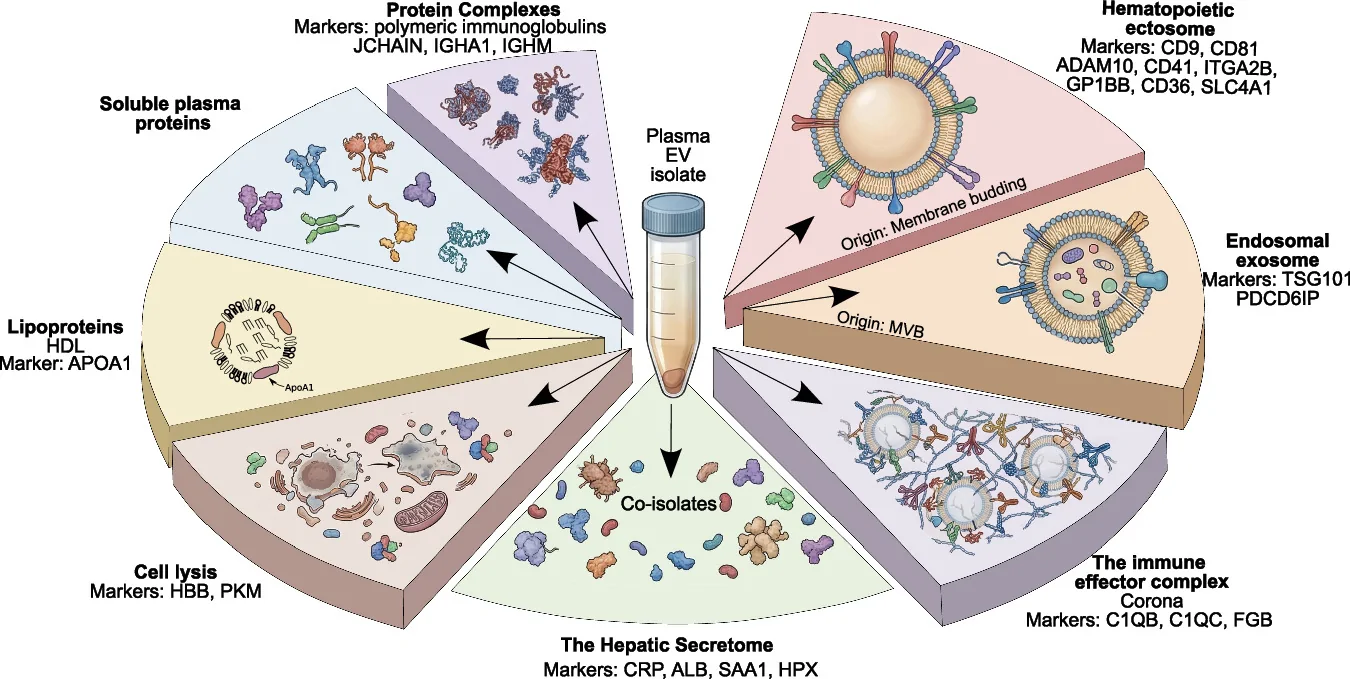
The modular architecture of the plasma EV isolate. The central schematic represents the bulk plasma EV isolate, often perceived as a homogeneous sample. The sections illustrate the biological deconvolution of this isolate into distinct variance components, as resolved by our co-abundance analysis. The Hematopoietic Ectosome module (Major Fraction) captures the bulk of the tetraspanin signal (CD9, ADAM10) and is derived from platelet/RBC membrane shedding. This is structurally distinct from the Endosomal Exosome module (Fraction), which contains the ESCRT machinery (TSG101, ALIX). Additional slices represent the Functional Matrix of co-isolated components, including the Immune Effector Complex (C1q, IgG), the Hepatic Secretome (CRP, Albumin), and Lipoproteins (ApoA1). Biomarker specificity requires resolving these independent biological sources rather than treating the isolate as a singular entity.

We therefore propose replacing binary EV vs. non-EV lists with a component-based reporting framework, as summarized in Fig. 8. Because the plasma matrix and electrostatic corona cannot be completely eliminated, isolation efficacy should no longer be defined by the absolute absence of non-vesicular proteins. Instead, success must be evaluated by the relative enrichment of the vesicular modules compared to these unavoidable, physically associated background components (e.g., lipoproteins, complement, and immunoglobulins). Future plasma EV studies should quantify the relative abundance of at least these eight biological modules to establish the detailed characterization of the plasma EV samples. By decomposing the hepatic background and hematopoietic-derived EV networks from the endosomal signal, this atlas provides the necessary roadmap for discovering high-specificity biomarkers in liquid biopsy. To actively target and uncover these tissue-specific vesicular populations, we propose two distinct, forward-looking bioinformatic strategies based on our findings. First, an in silico background subtraction approach: the eight ubiquitous modules defined in this study (e.g., the platelet, erythrocyte, lipoprotein, and complement clusters) can be utilized as a standardized bioinformatic exclusion matrix. By computationally subtracting the mass spectrometry signal of these dominant background modules from new clinical datasets, future studies can artificially deepen analytical sensitivity to reveal underlying, low-abundance tissue-specific EV signatures. Second, the development of supervised deconvolution architectures: while our study utilized unsupervised clustering, which naturally resolves only the most abundant, ubiquitous signals, future analytical pipelines should transition to supervised bioinformatic deconvolution, analogous to CIBERSORT algorithms^33^ used in bulk tissue transcriptomics. By training machine-learning models on pure, isolated tissue-EV signatures (e.g., brain, liver, or tumor-derived secretomes), it will be possible to computationally extract and quantify the fractional contribution of these rare populations from within the complex bulk plasma EV signal. It is crucial to note that while our co-variance analysis robustly defines co-fractionating statistical networks, computational clustering alone cannot definitively prove that these markers co-reside on the exact same physical lipid bilayer. Absolute proof of shared physical origin within these defined modules will ultimately require orthogonal biochemical validation, such as single-vesicle flow cytometry or high-resolution immuno-affinity isolation.

In summary, the analysis presented here challenges the prevailing view of the plasma EV proteome as a homogeneous entity or mainly a heterogeneous vesicle entity. By defining the physical drivers of EV isolation and subsequently decomposing the variance of 515 clinical samples, we demonstrate that the reproducible plasma EV isolate is not a single biological entity. Instead, it is driven by three overarching mechanistic origins, biophysical mass-action, macromolecular co-isolation, and phlebotomy-induced trauma, which further stratify across the clinical population into at least eight distinct biological modules. Our data reveal that high-abundance proteins frequently cited as EV markers, including acute-phase reactants (CRP, SAA), complement factors (C1q, C3), and coagulation regulators, segregate into the Hepatic Secretome and Immune Effector clusters. These proteins represent a reproducible co-isolated plasma fraction driven by solubility and surface adsorption, distinct from vesicle biogenesis. Therefore, we propose that the observed fluctuation in these markers more accurately reflects systemic liver responses or immune complex formation, rather than direct vesicular regulation. Furthermore, we resolve the lineage attribution of canonical EV markers. The clustering of CD9 and ADAM10 with platelet and erythrocyte membranes (Cluster 7, Supplementary Data 9 and Cluster 5, Supplementary Data 10) suggests that these targets primarily monitor the total burden of blood cell-derived EVs. In contrast, the distinct segregation of TSG101 and ALIX (Cluster 8) offers a specific metric for endosomal machinery that is independent of the bulk membrane background. Ultimately, this modular stratification transcends binary classifications of vesicular versus non-vesicular proteins. By mapping proteins to their specific biological source, whether the sub-membrane cortex, the lipid raft, or the soluble hepatic background, we provide a roadmap for high-resolution biomarker discovery that is robust to the inherent complexity of *human* plasma.

## Methods

### Systematic search and selection of public plasma EV proteomics datasets

#### Search strategy

Keywords used to query the PRIDE repository included: “plasma”, “extracellular vesicles”, “exosomes”, “serum”, and “*human*”. Initially, only datasets utilizing label-free quantitation and data-dependent acquisition (DDA) were maintained for the core meta-analysis. Data from the following accessions were retrieved: MSV000094307, PXD002668, PXD004420, PXD010404, PXD015283, PXD016293, PXD018301, PXD018337, PXD020629, PXD021718, PXD024072, PXD024216, PXD026483, PXD028515, PXD028885, PXD032767, PXD036735, PXD042379, and PXD042422. To orthogonally validate our findings across different mass spectrometry acquisition modes, additional label-free Data-Independent Acquisition (DIA) datasets from Wu et al. PXD042947^6^ and Suresh et al. PXD058948^18^ were retrieved and incorporated into the comparative analysis.

#### Inclusion criteria

For the primary meta-analysis, datasets were retained if they contained ≥3 raw files, consisted exclusively of *human* samples, were compatible with label-free quantification, and utilized DDA. The supplementary validation datasets (Wu et al.^6^, Suresh et al.^18^) were required to meet the same biological and sample criteria but utilized DIA.

#### Exclusion criteria

Datasets using stable isotope-based quantitation (e.g., SILAC, TMT) were excluded, as they are technically challenging to harmonize directly with label-free datasets. Initially, DIA datasets were also excluded to ensure strict technical uniformity across the primary meta-analysis computational pipeline. However, as noted above, representative DIA datasets were subsequently included as a validation cohort to demonstrate the cross-platform robustness of our analysis.

#### Final dataset composition

The core DDA search resulted in a comprehensive dataset of 515 LC-MS/MS runs covering a broad range of plasma EV isolation methods. These methods included differential centrifugation (DC), differential ultracentrifugation (DUC), differential centrifugation followed by size exclusion chromatography (DC_SEC), density gradient ultracentrifugation (DGUC), differential ultracentrifugation followed by sucrose gradient (DUC_SUC), size exclusion chromatography (SEC), ExoRNeasy (Qiagen Italia, Milan, Italy), polymer-based precipitation followed by density gradient ultracentrifugation (PP_DGUC), polymer-based precipitation followed by size exclusion chromatography (PP_SEC), and PRotein Organic Solvent Precipitation (PROSPR). This core dataset of 515 runs was further supported by the targeted inclusion of the aforementioned DIA datasets.

### Unified raw data processing (MaxQuant workflow)

LC-MS data from previous studies based on *human* EV samples were downloaded from ProteomeXchange Consortium website^5,14,34–38^ (24th of March 2024). The data were analyzed by MaxQuant version (2.1) to ensure uniformity. The *human* UniProt FASTA database was used for database dependent search (UP000005640). Carbamidomethyl cysteine was specified as the fixed modification. Variable modifications included methionine oxidation, and N-terminal protein acetylation. Quantification was performed using label-free quantification (LFQ) (Fast LFQ enabled, and min. ratio count = 2) and intensity based quantification (iBAQ). The False Discovery Rate (FDR) threshold was set to 1% at peptide and protein levels. Reverse and contaminant matches were filtered out in R. To maintain the integrity of detection statistics, missing intensity values (NaN) were imputed with zero. This conservative approach was selected to strictly preserve the presence or absence information inherent in Data-Dependent Acquisition (DDA) mass spectrometry. Unlike probabilistic imputation methods (e.g., KNN or min-draw) that generate synthetic low-abundance values, zero-imputation ensures that the definition of the core plasma EV proteome remains restricted to proteins with genuine, reproducible spectral evidence. Consequently, proteins with sporadic detection profiles are penalized in mean-abundance calculations, ensuring that the downstream correlation analysis focuses exclusively on the robust, high-abundance fraction of the isolate. Finally, rank normalization was performed and the data filtered to maintain proteins detected in 50% of all LC-MS runs.

### Construction of high-resolution reference proteomes for non-vesicular components

To accurately deconvolute the plasma EV proteome, we established a compendium of high-depth reference datasets representing the primary non-vesicular sources of variance in plasma (e.g., platelets, erythrocytes, lipoproteins, and soluble immune complexes). This reference library was constructed through a multi-stage literature mining and manual curation pipeline designed to maximize specificity. Crucially, to avoid the circularity inherent in utilizing literature-derived EV databases, which often inherently contain co-isolated plasma proteins, our reference signatures for these functional networks (e.g., platelets, erythrocytes, liver) were derived strictly from primary cellular and tissue proteomics studies. First, we defined a panel of anchor proteins (Table 1) to serve as definitive, high-consensus markers for each non-vesicular biological compartment and EV markers. These anchors were selected based on their established utility in clinical diagnostics and cell biology as lineage-specific or compartment-specific indices (e.g., ITGA2B/CD41 for platelets, APOA1 for HDL, HBB for hemolysis). Using these anchor proteins as seeds, we mined high-resolution proteomic studies (studies referenced in Table 1) to compile a comprehensive associated proteome for each compartment, which consists of proteins abundant in these compartments (Supplementary Data 1). This dataset encompasses all high-abundance proteins reported to functionally interact with or co-purify with the respective components. This list captures the broad interactome of plasma co-isolates, including both integral component proteins and transiently associated binding partners. This list was then further processed. To ensure quantitative rigor in downstream analyses in the “Results” section, we applied a strict manual curation filter to the associated proteome to generate a functional reference set (Supplementary Data 3). This step was critical to remove proteins that are merely associated (e.g., ubiquitous chaperones, non-specific matrix proteins) and retain only those with a clear functional annotation defining them as integral to the biological component. Criteria for inclusion in Supplementary Data 3 required obligate components of the macromolecular complex (e.g., *C1q* subunits for the complement complex). This curated Supplementary Data 3 served as the definitive gene set for the quantitative enrichment analyses presented in the results sections, ensuring that calculated abundance metrics reflected specific biological co-isolation burdens rather than generic protein noise.

### Calculation of relative co-isolate module burden

To account for varying overall EV yields across different isolation methodologies and to provide a standardized metric for preparation purity, we developed the “Relative Co-isolate Module Burden”. This normalization strategy utilizes canonical EV structural proteins as an internal reference, addressing the need for a robust alternative to rank-based normalization.

First, the baseline EV signal (denoted as the “mean ISEV EV signal”) was established for each dataset by calculating the mean quantitative iBAQ abundance of core EV markers recommended by the International Society for Extracellular Vesicles (ISEV) guidelines (e.g., CD9, CD63, CD81, SDCBP/Syntenin-1, TSG101, and PDCD6IP/ALIX) that were detected in the respective sample. Second, the “mean module signal” was determined by calculating the mean quantitative abundance of the identified plasma co-isolate/corona proteins.

To compute the final metric, a pseudocount of 1 was added to both mean values prior to log_2_-transformation to prevent undefined mathematical operations (e.g., log of zero or division by zero) in cases of missing or zero-value protein quantifications. The Relative Co-isolate Module Burden was thus defined by the following formula:

$${\rm{Relative}} \, {\rm{Co}}{\mbox{-}} {\rm{isolate}} \, {\rm{Module}} \, {\rm{Burden}} = \log_{2}({\rm{mean}} \, {\rm{module}} \, {\rm{signal}} + 1) {/} \\ \log_2({\rm{mean}} \, {\rm{ISEV}} \, {\rm{EV}} \, {\rm{signal}+1})$$

By dividing the log-transformed co-isolate abundance by the log-transformed ISEV marker abundance, this metric yields a standardized index. A higher burden score indicates a greater proportion of plasma co-isolates relative to EV structural proteins, thereby enabling direct cross-platform and cross-methodological comparisons of EV preparation purity independent of absolute instrument sensitivity.

### Plasma proteome abundance and distribution analysis

To evaluate the physiological abundance distribution of the core plasma EV proteome, baseline *human* plasma protein concentrations were retrieved from the comprehensive *human* plasma PeptideAtlas database (build 2025-08). Abundance values for both the background physiological plasma proteome and the empirically defined core plasma EV proteome were log_10_-transformed prior to analysis. To visualize concentration-driven sampling bias, rank-abundance curves were constructed by sorting the entire detectable background plasma proteome by descending log_10_ abundance. Proteins comprising the core plasma EV proteome were subsequently mapped onto this curve and stratified by their assigned co-isolation module signatures. Differences in the abundance distributions between the core EV proteome and the background plasma proteome were quantified using empirical cumulative distribution functions (ECDF). Statistical significance between the two distributions was calculated using a two-sided, two-sample Kolmogorov–Smirnov test (stats::ks.test in R). All associated visualizations, including the rank-abundance scatter plots and ECDF distributions, were generated using the ggplot2 package in the R statistical computing environment.

### Calculation of abundance-weighted isoelectric points (pI)

To evaluate the biophysical and electrostatic constraints governing protein co-isolation, abundance-weighted isoelectric points were calculated for both the core plasma EV proteome and the synthetic nanoparticle coronas. Quantitative nanoparticle corona data were obtained from the PRIDE repository (dataset identifier PXD017052). Theoretical isoelectric points for all identified proteins were derived using standard UniProt amino acid sequence annotations.

To accurately reflect the mass-action bias driven by highly abundant plasma proteins, the theoretical pI of each protein was weighted by its relative stoichiometric abundance within the respective sample. Prior to weighting, log_2_-transformed quantitative values (Log_2_ iBAQ) were exponentiated to restore a linear abundance scale. The abundance-weighted mean pI for each sample was subsequently calculated by taking the sum of the products of each intersecting protein’s theoretical pI and its linear abundance, divided by the total linear abundance of all valid proteins within that sample. Proteins lacking definitive sequence data or containing non-canonical amino acids that prevented theoretical pI calculation were excluded from the weighting formulation.

### Proteomic correlation and stoichiometric alignment

Stoichiometric similarity between the core plasma EV proteome and the distinct synthetic nanoparticle coronas was evaluated using Spearman’s rank-order correlation (*ρ*). This non-parametric approach was specifically selected to mitigate the mathematical distortion typically caused by extreme abundance outliers (e.g., albumin and immunoglobulins) inherent to plasma proteomics data.

To prevent nanoparticles with highly restricted proteomic sampling (low overlap) from yielding artificially high correlation scores, a custom coverage-weighted Spearman’s *ρ* was calculated. This unified metric penalizes limited proteomic capture while rewarding comprehensive stoichiometric alignment. The coverage-weighted index was defined as the standard Spearman correlation coefficient of the intersecting proteins multiplied by the coverage fraction (calculated as the number of matching proteins divided by the total number of proteins comprising the core EV proteome). The statistical significance (two-sided *P*-values) of the monotonic relationships was determined utilizing standard asymptotic approximations. All data integration, reshaping, and statistical calculations were executed programmatically in R.

### Cellular origin profiling via platelet-derived EVs

To evaluate the contribution of ex vivo platelet degranulation to the core plasma EV proteome, quantitative proteomic profiles of agonist-induced platelet-derived EVs (pEVs) were obtained from a recently published reference dataset (ProteomeXchange/MASSIVE identifier: MSV000093007). This dataset encompasses EVs released by isolated washed *human* platelets stimulated with four distinct physiological agonists: adenosine diphosphate (ADP), collagen, thrombin, and a dual stimulation of thrombin/collagen.

To establish quantitative cellular reference profiles, the label-free quantification (iBAQ) intensities across all biological replicates for each specific agonist condition were averaged. Prior to averaging, the data matrices were subjected to strict filtration to prevent imputation artifacts; string-based missing value notations (e.g., “NA”, “NaN”) and exact zero values inherent to data-dependent acquisition dropouts were coerced to logical missing values (NA). The stoichiometric similarity between the core plasma EV proteome and each of the four specific pEV activation states was subsequently evaluated utilizing the custom Coverage-Weighted Spearman’s rank correlation described above.

### Comprehensive decomposition of the plasma EV core

To definitively parse the biophysical and cellular origins of the plasma EV isolate, a multi-dimensional quantitative intersection was performed utilizing comprehensive pan-corona and pan-platelet reference models.

A pan-corona reference profile was generated to capture the maximal biophysical binding potential of standard mass-action. This was calculated by extracting the maximum quantitative abundance for each protein across all 11 distinct synthetic nanoparticle chemistries detailed in the Blume dataset. Concurrently, a pan-platelet cellular reference profile was generated to capture the complete spectrum of ex vivo vesiculation by extracting the maximum quantitative abundance for each protein across all four agonist-induced pEV states (ADP, collagen, thrombin, and thrombin/collagen).

The core plasma EV proteome was subsequently mapped against these maximal reference profiles and categorically partitioned into four distinct functional modules based on confirmed quantitative overlap (defined strictly as an abundance >0 in the respective dataset):

- Pan-Corona Driven: Proteins mapped exclusively to the pan-corona reference, representing generalized biophysical co-isolation.
- Vesicle Driven: Proteins mapped exclusively to the pan-platelet reference, representing cellular cargo likely driven by ex vivo platelet activation.
- Shared: Proteins with confirmed quantitative abundance in both the pan-corona reference and pan-platelet reference datasets.
- Unassigned (Unique to plasma EV Core): Proteins completely absent from both generalized synthetic coronas and platelet-derived EV reference.

For visual stoichiometric decomposition, the core plasma EV proteome was ranked by descending Log_10_ abundance, and the individual proteins were mapped onto the rank-abundance distribution according to their assigned functional module. All data integration, filtering, and categorical mapping were programmatically executed in R.

### Statistics and reproducibility

This study consists of a secondary computational meta-analysis of 515 *human* plasma EV proteomes derived from publicly available datasets. As a retrospective in silico study, traditional experimental replicates are not applicable; however, reproducibility of all statistical analyses, variance deconvolutions, and visualizations is ensured by providing the complete, documented R code and intermediate data files as Supplementary Software. All statistical analyses were performed within the R statistical programming environment (V4.2.2). To map the variance structure of the plasma EV proteome, rank-normalized iBAQ intensities were subjected to pairwise correlation analysis and unsupervised hierarchical clustering. Correlation and Clustering Methodology: To identify protein modules exhibiting consistent co-isolation patterns across the cohort, we calculated a pairwise Pearson correlation matrix for all proteins in the core and candidate datasets. Unsupervised agglomerative hierarchical clustering was performed on the correlation matrix using Euclidean distance as the dissimilarity metric and Ward’s minimum variance method (ward.D2) to minimize within-cluster variance. Heatmap Visualization and Cluster Definition: Clustered data were visualized using the pheatmap R package (V1.0.12). The optimal number of clusters k was determined by evaluating the dendrogram fusion heights (linkage distance). Inspection of the scree plot of linkage distances revealed a distinct plateau rather than a sharp elbow, indicating stable separation of the proteome into at least eight primary variance components. Consequently, the dendrogram was cut at *k* = 8 using the cutree function (R stats package). Module Significance: Consistent with established benchmarks for plasma proteomics (Geyer et al.^10^), correlation modules exhibiting a mean internal Pearson correlation coefficient of *r* > 0.5 were defined as significant co-isolation units. This threshold distinguishes robust physical co-complexes and co-isolating particles from stochastic background noise.

### Visualization

Schematic illustration (Fig. 8) was conceptualized with the assistance of Gemini and manually redrawn in Inkscape (v1.1) to ensure scientific accuracy and alignment with heatmap data generated in this study.

## Supplementary information

Transparent Peer Review file

Supplemental Figures

Description of Additional Supplementary Files

Supplementary Data 1

Supplementary Data 2

Supplementary Data 3

Supplementary Data 4

Supplementary Data 5

Supplementary Data 6

Supplementary Data 7

Supplementary Data 8

Supplementary Data 9

Supplementary Data 10

Supplementary Software

## Data Availability

All datasets analyzed during the current study are publicly available. The mass spectrometry proteomics datasets can be accessed in the ProteomeXchange^14^ and PRIDE^15^ databases under accession numbers: MSV000093007, PXD017052, MSV000094307, PXD002668, PXD004420, PXD010404, PXD015283, PXD016293, PXD018301, PXD018337, PXD020629, PXD021718, PXD024072, PXD024216, PXD026483, PXD028515, PXD028885, PXD032767, PXD036735, PXD042379, and PXD042422. All other data supporting the findings of this study are available within the paper and its supplementary information files.

## Code availability

All software used for data processing and analysis in this study are publicly available R packages. The analyses were performed in the R statistical computing environment (R version 4.2.2). The specific R packages used to process the data and generate the figures include ggplot2, dplyr, pheatmap, and tibble. The custom R scripts utilized to perform the analyses and generate the main text Figures in this manuscript are provided as Supplementary Software 1. The source data underlying the clustering analyses, correlation networks, and resulting main text figures are provided in Supplementary Data 1–10 (see “Data Availability” section) and R code under section code availability. Code file “Figure3_Abundance.R” will load relevant data and reproduce Fig. 3. Code file “Figure4_RelativeModuleBurden.R” will load relevant data and reproduce Fig. 4. Code file “Figure5_RankAbundance_CDF_plasma_EV_plasma.R” will load relevant data and reproduce Fig. 5. Code file “Figure6_decompose_EV_Core.R” will load relevant data and reproduce Fig. 6. Code file “Figure7_CorHeatmap.R” will load relevant data and reproduce Fig. 7. Code file “FigureS5_RelativeModuleBurden_DDA_Methods.R” will load relevant data and reproduce Figure S5. Code file “Figure S6_RelativeModuleBurden_DIA_Suresh_Methods.R” will load relevant data and reproduce Figure S6. Code file “Figure S7_RelativeModuleBurden_DIA_Wu_Methods.R” will load relevant data and reproduce Figure S7. Code file “TableS5_Cor_EVcore_Blume.R” will load relevant data and reproduce result data in Supplementary Data 5.
