# Supplementary material for: Analysis of 515 human proteomes defines plasma extracellular vesicle core composition, corona and coisolated networks
Source: Commun Biol. 2026 Oct 30;9:1402. doi: 10.1038/s42003-026-11038-6 (PMC13722686; doi:10.1038/s42003-026-11038-6)
Supplement: Supplementary file 1 [file 42003_2026_11038_MOESM1_ESM.pdf]

nature portfolio

Peer Review File

Analysis of 515 human proteomes defines plasma extracellular vesicle core composition, corona and coisolated networks

Corresponding Author: Dr Rune Matthiesen

This file contains all reviewer reports in order by version, followed by all author rebuttals in order by version.

Version 0:

Reviewer comments:

Reviewer #1

(Remarks to the Author)
This manuscript presents a large-scale meta-analysis of 515 human plasma extracellular vesicle (EV) proteomics datasets with the aim of defining a core plasma EV proteome and resolving its underlying biological components. The study is timely and addresses a central challenge in the EV field, namely the interpretation of plasma-derived EV preparations in the context of co-isolated plasma components. The scale of the dataset and the effort to harmonize multiple studies are notable strengths. However, several conceptual and methodological concerns limit the strength of the conclusions and require substantial revision.

- The central conclusion that the reproducible plasma EV proteome is dominated by non-vesicular components appears overstated. The analysis does not adequately distinguish between intravesicular cargo, surface-associated proteins (e.g., protein corona), and truly co-isolated soluble components. Given the well-established interactions between EVs and plasma proteins, many molecules classified here as “contaminants” may in fact be EV-associated and biologically relevant. The authors should therefore avoid a binary EV versus non-EV classification and instead adopt a more nuanced framework reflecting different modes of association.

- The interpretation of canonical EV markers is also problematic. The conclusion that tetraspanins such as CD9 and CD81 primarily reflect hematopoietic ectosomes is not sufficiently supported by the data. Co-clustering with platelet and erythrocyte proteins indicates enrichment from hematopoietic sources, but does not demonstrate vesicle biogenesis pathway or exclude their presence in other EV subtypes. Similarly, the claim that ESCRT-associated proteins define a minor EV population is likely influenced by detection bias in mass spectrometry, as these proteins are typically of low abundance. The authors should temper these conclusions and clearly acknowledge the limitations of abundance-based inference.

- From a methodological perspective, the integration of datasets from multiple studies using diverse isolation methods raises concerns about batch effects and technical variability. The observation that samples cluster by study suggests that technical factors may significantly influence the results, yet no formal batch correction or variance partitioning is presented. Additionally, the use of zero-imputation for missing values in data-dependent acquisition proteomics is problematic and likely biases the analysis toward highly abundant plasma proteins. The definition of the “core proteome” based on a ≥50% detection threshold further reinforces this bias and may not accurately reflect EV biology.

- The interpretation of correlation-based clustering as evidence of distinct biological modules should be treated with caution. Co-abundance does not necessarily imply co-isolation or shared origin, and alternative explanations such as shared detectability or technical co-variation should be considered. The contamination reference sets derived from the literature may also introduce circularity, as many published EV datasets already contain co-isolated plasma proteins.

While the dataset and analytical approach are potentially valuable, the manuscript would benefit from a more cautious interpretation of the results, improved handling of methodological limitations, and a revised conceptual framework that better reflects the complexity of plasma EV preparations. I recommend major revision prior to further consideration.

Reviewer #2

(Remarks to the Author)
This meta-analysis study presents a large-scale proteome-centric analysis of 20 different plasma EV datasets and proposes that EV isolates comprise reproducible co-isolated biological modules rather than pure vesicle populations. This concept is not new, but the analytical insights are. The work addresses a central issue in human EV biology, namely the extent to which plasma EV preparations reflect bona fide vesicles versus co-isolated plasma components. The study also identifies eight co-isolating protein modules. While indicant recent human multi-omic studies have recently been reported, this study actually complements and adds to further these concepts. The scale of integration and the attempt to derive consensus proteomic structure across studies are commendable. However some key limitations exist, including the sole computational nature of the study, terminology, and several of its terms and analysis framework

A query in the analysis is that the studies used in the literature search and analysis section are mainly cell based EV studies and not plasma/biofluid. This presents significant limitations with how the field of EVs is now understanding EVs from plasma as distinct from cell-derived origin. Therefore direct links of defining and validating EV and nonEV markers is dependent on their source material.

#1. One questions the use of the term contamination signatures. These should be removed given the nature of the sample matrix – you will never completely remove these, but rather focus on enrichment. For example complement proteins and immunoglobulins may bind EV surfaces, and Lipoproteins may physically associate with EVs. The key factor here is enrichment relative to the presence of these multiple biological module factors. This needs to be integrated throughout

#2. Blood processing and plasma pre-analytical variation. Does the preparation, storage time of plasma impact the core proteome? These variables need to be introduced for the 20 studies profiled and built into the analysis framework

#3. Consistency. In the study 344 proteins are reported across studies at identification rate >50%. When this is increased to 80%, do these key markers still remain dependent on the type of approach to isolate and proteomically analyse? Why 50%? Isn't this just a proteomic dynamic range factor rather than the core composition of an EV? Is this the same peptides being identified across different studies that comprise this core atlas? How was the data overall normalised or was this a single batch analysis framework? How were batch corrections and random effects modelling performed? This needs to be rigorously built in for the formal analysis and figures.

#4. The composition of plasma EVs. While significant focus on the plasma EV itself, what about other co-isolated protein modules. Can these be compared to large scale plasma proteome efforts? In context of Rai et al as described, can the non-EV components identified consistent in this study from plasma also be contrasted here. Are these just contributed by the protein corona where knowledge in this at a proteome level interacting with various nanoparticles is being presented, eg Blume et al 2020.

#5. A key finding is identification of CD9, CD81, and ADAM10 cluster with platelet and erythrocyte proteins, suggesting that their signal in plasma largely derives from hematopoietic ectosomes rather than generic EVs. Can other studies be included here to support such statements. What about platelet activation markers or RBC shedding markers from proteomics across the same 20 studies? What about platelet activation EV release factors at a proteome level (e. Moon 2024). Co-variation reflects shared cellular origin, not misannotation. This wording and the way in which this deconvolution is being performed needs to be improved for accuracy and clarity

#6. Check spelling and details in figures and throughout. E.g., figure 4-5; platlets

#7. What was the inclusion criteria for these 515 proteomes? It seems just a search parameter focus was used – and that the detail in the stringency of obtaining an EV was not given? Some are poor quality based on coverage/depth, and correlation with their EV. A clear focus on why these 515 (and 20 studies) specifically is needed. Why not focus just on the 50% of study proteomes that are being used to generate the core and comparative elements of the study? Are the remaining 50% EV at all, or just plasma proteome sets? This detail need careful focus, given the narrative is for the analysis and interpretation of EVs.

#8. The exclusion of DIA centric data is a major limitation. The expansion in DIA with EV proteome studies needs to be factored in. Not a written explanation but integration of various DIA-human EV plasma sets to validate the core EV elements and other findings. The use of DIA is more prevalent in the past 2-3 years, and has overcome significant limitations in acquisition, coverage and quantitative accuracy.

Reviewer #3

(Remarks to the Author)
The distinction between EVs and contaminants, found in almost all purification strategies, is a critical issue in the EV field. This is all the more true for complex biological fluids such as blood plasma, which is an important source of biomarkers. Performing a meta-analysis from a large set of proteomic studies, the authors first identify a reproducible core proteome of 350 proteins based on their frequency of detection (>50%) in the LC-MS runs, and then, characterize the content of this core proteome by clustering strategies. They demonstrate that this core proteome mainly consists in contaminating non-EV proteins but further refine its composition into 8 biological modules, thus replacing the binary EV non-EV subdivision. An important finding is the clustering of ectosome (plasma membrane signature) markers, but not exosome markers (endosomal

signature), with the hematopoietic lineage, which is highly relevant when searching for biomarkers.

I will not comment on the bioinformatic tools used in this study because of lack of expertise but restrict my comments to the global strategy and the conclusions drawn by the authors.

It is classically reported that the nature of contaminants mainly depends on the purification strategy, as shown also in sup Fig2. Almost 60% of the individual runs analysed in this study derived from only 2 different purification strategies (fig1a), and 20% likely issue from a single lab (RNeasy purification). It challenges the definition of the core proteome which is based on the frequency of detection of proteins in all individuals MS-runs without weighting the data. For instance, the detection frequency of the core proteins relative to the number of studies gives a different picture (Fig3b), often being under the 50% threshold. How the different clusters identified by the authors distribute depending on the purification strategy might be informative for the selection of the most appropriate one.
The authors nicely confirm the abundance of non-EV contaminants in EV preps (although now widely accepted in the field) but might more deeply comment their findings with those of other previous studies (e.g. Vallejo 2023).
From the literature, tissue-derived EVs represent at maximum few % of plasma EVs. The fact that “the EV proteome signal in bulk plasma is primarily driven by the burden of hematopoietic ectosomes rather than generic tissue-derived exosome secretion” is thus not unexpected. This has to be quoted . Focusing on the core proteome might hinder the detection of this minor component of the EV proteome. From the authors findings, are alternative and more directed bioinformatic strategies could be consider to target specifically this population?

Version 1:

Reviewer comments:

Reviewer #1

(Remarks to the Author)
I would like to thank the authors for the detailed and rigorous answers for each point of my previous reviewing. This reviewing step has been led in a state-of-the-art manner and answered to all my questions or remarks, and the quality of the manuscript is now indiscutable.

Reviewer #2

(Remarks to the Author)
all key areas and queries raised have been addressed and content in the revised manuscript updated. well done and happy to proceed here

Reviewer #3

(Remarks to the Author)
In this revised version of the manuscript, the authors provide a valuable set of new analyses, including the ones I suggested. May be the most instructive is the inclusion of DIA data which appear to corroborate the DDA analysis and be more robust across purification methods (figure S6, abbreviation of each method should be defined, e.g. TEI), also reflecting the nature of the starting material (Wu et al study). May be the DDA/DIA comparison might be further comment in the discussion and one figure included in the main text. The inclusion of the corona composition based on Blume et al. study also brings some new insights although reducing EV binding properties of corona proteins just to their pI looks rather simplistic and could be nuanced in the discussion.
Beyond these suggestions. The new data and answers provided by the authors justified the acceptance of the manuscript.

Open Access This Peer Review File is licensed under a Creative Commons Attribution 4.0 International License, which permits use, sharing, adaptation, distribution and reproduction in any medium or format, as long as you give appropriate credit to the original author(s) and the source, provide a link to the Creative Commons license, and indicate if changes were made.
In cases where reviewers are anonymous, credit should be given to 'Anonymous Referee' and the source.
The images or other third party material in this Peer Review File are included in the article's Creative Commons license, unless indicated otherwise in a credit line to the material. If material is not included in the article's Creative Commons license and your intended use is not permitted by statutory regulation or exceeds the permitted use, you will need to obtain permission directly from the copyright holder.
To view a copy of this license, visit https://creativecommons.org/licenses/by/4.0/

We thank the editor and the reviewers for the time, highly constructive feedback, and rigorous evaluation of our manuscript. We are grateful for the opportunity to submit this revised version. The reviewer comments and specific editorial directives have been invaluable in refining our theoretical framework and statistical methodologies.

Below, we have addressed each specific comment from the reviewers' point-by-point (R: Review, P: point).

Reviewer 1 (R1):
R1 P1: The central conclusion that the reproducible plasma EV proteome is dominated by non-vesicular components appears overstated. The analysis does not adequately distinguish between intravesicular cargo, surface-associated proteins (e.g., protein corona), and truly co-isolated soluble components. Given the well-established interactions between EVs and plasma proteins, many molecules classified here as contaminants may in fact be EV-associated and biologically relevant. The authors should therefore avoid a binary EV versus non-EV classification and instead adopt a more nuanced framework reflecting different modes of association.

AU: We thank the reviewer for this insightful comment and completely agree with the underlying point of view. The biological reality of plasma EVs is highly complex, and the strict binary classification of "vesicle" versus "contaminant" fails to capture phenomena such as secondary recruitment, indirect tethering, and the formation of a functional protein corona.
To address this critical point, we have comprehensively revised the manuscript to adopt the nuanced framework the reviewer suggests:

1. *Terminology update: We have removed the binary "EV vs. contaminant" language throughout the text. We now refer to these non-core modules as "co-isolates", or "non-EV components". We explicitly acknowledge in the revised Discussion (Page 17-18) that molecules in these modules may not be freely soluble artifacts, but rather biologically relevant, surface-associated corona proteins that interact with EVs in circulation.*

2. *Clarifying the Analytical Framework: While we agree with the concept of different spatial modes of association (intravesicular cargo vs. surface-associated corona), we must note that our high-throughput bulk proteomic dataset (N=515) was designed to identify statistical covariance across a population, rather than spatial topology within individual vesicles. Because our isolates were not subjected to topological mapping (e.g., differential permeabilization or protease protection assays), assigning our statistical clusters strictly to "lumenal", "membrane", or "corona" compartments would be speculative.*

3. *Reframing the Modules: Instead of spatial compartments, we have clarified that our deconvolution identifies independent variance components. For example, our data shows that the abundance of the "soluble immunoglobulin complexes" fluctuates independently from the core vesicular biogenesis machinery across the 515 patients. While these soluble immunoglobulin complexes proteins may physically travel on the surface of the EV (as a corona), their statistical independence warrants their classification as a distinct biological module rather than core intravesicular cargo.*

R1 P2: The interpretation of canonical EV markers is also problematic. The conclusion that tetraspanins such as CD9 and CD81 primarily reflect hematopoietic ectosomes is not sufficiently supported by the data. Co-clustering with platelet and erythrocyte proteins indicates enrichment from hematopoietic sources, but does not demonstrate vesicle biogenesis pathway or exclude their presence in other EV subtypes. Similarly, the claim that ESCRT-associated proteins define a minor EV population is likely influenced by detection bias in mass spectrometry, as these proteins are typically of low abundance. The authors should temper these conclusions and clearly acknowledge the limitations of abundance-based inference.

AU: We thank the reviewer for this critical and highly constructive feedback. We completely agree that we overstepped in equating statistical covariance with specific biogenesis pathways, and that inferring vesicle

population size purely from mass spectrometry (MS) abundance has limitations. We have revised the text to temper these conclusions and explicitly address MS detection biases.
Regarding the specific points raised:

1. Tetraspanins and Ectosomes: We agree that the co-clustering of CD9/CD81 with platelet and erythrocyte markers strongly indicates a hematopoietic source, but it cannot definitively prove an ectosomal biogenesis pathway or exclude their presence on other EV subtypes. We have removed the definitive claims tying these modules to "ectosomes" and now refer to this cluster more accurately as a "hematopoietic-derived EV network".

2. ESCRT and MS Detection Bias: The reviewer raises an excellent point regarding detection bias. While ESCRT components like TSG101 and ALIX are soluble/cytosolic-facing proteins, and thus do not suffer from the severe extraction and ionization biases typical of highly hydrophobic, multi-pass transmembrane proteins like tetraspanins, they are inherently low-copy-number proteins per vesicle. Therefore, a low MS signal for TSG101 may reflect its stoichiometry rather than a low number of total endosomal exosomes. Because ESCRT components are internal machinery with a low structural copy number per vesicle, their low MS signal cannot be used to reliably infer that endosomal exosomes are a minor population overall.

We have modified the Discussion (Page 18) to reflect this. We no longer claim endosomal exosomes are a "minor fraction". Instead, we focus solely on the robust mathematical observation: the ESCRT machinery forms a statistically distinct regulatory module that does not co-vary with the dominant hematopoietic tetraspanin signal in clinical plasma.

R1 and P3: From a methodological perspective, the integration of datasets from multiple studies using diverse isolation methods raises concerns about batch effects and technical variability. The observation that samples cluster by study suggests that technical factors may significantly influence the results, yet no formal batch correction or variance partitioning is presented. Additionally, the use of zero-imputation for missing values in data-dependent acquisition proteomics is problematic and likely biases the analysis toward highly abundant plasma proteins. The definition of the "core proteome" based on a ≥50% detection threshold further reinforces this bias and may not accurately reflect EV biology.

AU: We thank the reviewer for raising these important methodological points. We agree that in traditional comparative proteomics (e.g., differential expression analysis between two clinical states), batch correction and the avoidance of zero-imputation are standard best practices. However, because the primary objective of this meta-analysis was to evaluate the structural composition of the isolates across diverse methodologies, applying these standard corrections would have actively erased the very signals we aimed to measure. We respectfully provide the following clarifications, and have added a new sensitivity analysis to the revised manuscript to address these concerns robustly:

1. Batch Effects vs. Biological Modularity: The observation that samples cluster by study is not a confounding artifact; it is a central finding. Because different studies utilized different isolation methods (e.g., SEC vs. UC vs. Affinity), the batch effect is inextricably linked to the physical co-isolation of distinct biological networks (e.g., lipoproteins or immune complexes). Applying standard formal batch correction (such as ComBat or similar variance-removal algorithms) would mathematically force all isolation methods to look identical, effectively erasing the diverse "functional matrix" components we aimed to deconvolute. To account for variations in MS instrument sensitivity and loading amounts without destroying this critical inter-method variance, we utilized rank normalization, which provides a robust, non-parametric harmonization of the datasets.

2. The Rationale for Zero-Imputation: While zero-imputation is problematic when calculating subtle fold-changes, it is mathematically necessary when tracking presence/absence across diverse methodologies. In DDA proteomics of highly fractionated samples, missing values are predominantly Missing Not At Random (MNAR). For example, if a specific isolation method physically depletes a soluble protein, the failure of the mass spectrometer to detect it represents a true biological/technical zero, not stochastic missingness. If we had ignored missing values (treating them as NA) or imputed group means, proteins that were entirely

washed away in 400 samples but highly abundant in 100 samples would be mathematically treated as "universally present". Zero-imputation allows us to accurately penalize proteins that fail to be detected consistently across the field.

3. Sensitivity Analysis (new addition to the manuscript): We acknowledge the reviewer's concern regarding the ≥ 50% threshold and potential bias toward high-abundance proteins. To empirically demonstrate that our modular architecture is not an artifact of zero-imputation or this specific threshold, we have performed a comprehensive sensitivity analysis (See new Supplementary Fig. S4). For example, while applying stricter 70% and 90% thresholds results in significantly fewer proteins being retained in the EV core, it does not alter our fundamental finding: the EV signature consistently exhibits a lower apparent abundance than the majority of the co-isolate signatures. We believe this added sensitivity analysis explicitly addresses the reviewer's concerns and confirms the stability of our analytical framework.

R1 P4: The interpretation of correlation-based clustering as evidence of distinct biological modules should be treated with caution. Co-abundance does not necessarily imply co-isolation or shared origin, and alternative explanations such as shared detectability or technical co-variation should be considered. The contamination reference sets derived from the literature may also introduce circularity, as many published EV datasets already contain co-isolated plasma proteins.

AU: We thank the reviewer for highlighting these critical conceptual boundaries. We completely agree that circularity is a issue in EV informatics, and we appreciate the opportunity to clarify a crucial methodological detail that addresses this exact concern.
1. Eliminating Circularity in Reference Sets: The reviewer rightfully points out that deriving contaminant or co-isolation signatures from published EV datasets would introduce circularity, as those databases are inherently noisy. However, our methodology was explicitly designed to avoid this trap. Our reference signatures were NOT derived from EV studies. As detailed in the Method section, our functional network signatures were constructed exclusively from direct cellular and tissue-level proteomics (e.g., whole purified platelets, primary erythrocyte proteomics, and human liver tissue datasets). Because these signatures are anchored in the primary cellular source rather than downstream EV isolates, there is zero circularity in using them to identify non-vesicular, tissue-specific networks co-fractionating in plasma. We recognize that this distinction was not emphasized strongly enough in the original text, and we have now significantly clarified the origin of these reference sets in the Method sections to prevent future reader confusion (Page 22).

2. Co-abundance, Technical Variation, and Co-isolation: We agree with the premise that simple co-abundance does not definitively prove shared biological origin. However, in the context of physical separation techniques across 515 diverse samples, robust statistical covariance is the empirical signature of co-fractionation (what we term "co-isolates"). Regarding technical co-variation (e.g., shared detectability or MS loading artifacts), this is precisely why our pipeline utilizes rank normalization prior to clustering. By evaluating the relative rank of proteins within each MS run rather than raw intensities, we effectively neutralized global technical co-variation. The resulting clusters therefore represent independent physical behavior, modules of proteins that isolate together through physical separation matrices independently of the core vesicular machinery.

We have added a clarifying paragraph to the Discussion (Page 19) explicitly acknowledging that while clustering defines co-fractionating networks, absolute proof of shared physical origin requires orthogonal biochemical validation.

R1 P5: While the dataset and analytical approach are potentially valuable, the manuscript would benefit from a more cautious interpretation of the results, improved handling of methodological limitations, and a revised

conceptual framework that better reflects the complexity of plasma EV preparations. I recommend major revision prior to further consideration.

AU. We sincerely thank Reviewer 1 for rigorous, expert, and highly constructive evaluation of our manuscript. We fully agree that our original binary framing did not do justice to the biological complexity of the plasma EV environment. By integrating the reviewer's feedback, specifically by adopting a nuanced 'co-isolate/corona' framework, directly addressing the limitations of MS abundance inferences, and providing comprehensive sensitivity analyses, we believe the manuscript has been fundamentally strengthened. We are grateful for the reviewer's time and insight, which have greatly improved the quality and accuracy of this work.

Reviewer 2:

R2 P1: This meta-analysis study presents a large-scale proteome-centric analysis of 20 different plasma EV datasets and proposes that EV isolates comprise reproducible co-isolated biological modules rather than pure vesicle populations. This concept is not new, but the analytical insights are. The work addresses a central issue in human EV biology, namely the extent to which plasma EV preparations reflect bona fide vesicles versus co-isolated plasma components. The study also identifies eight co-isolating protein modules. While indicant recent human multi-omic studies have recently been reported, this study actually complements and adds to further these concepts. The scale of integration and the attempt to derive consensus proteomic structure across studies are commendable. However some key limitations exist, including the sole computational nature of the study, terminology, and several of its terms and analysis framework.

A query in the analysis is that the studies used in the literature search and analysis section are mainly cell based EV studies and not plasma/biofluid. This presents significant limitations with how the field of EVs is now understanding EVs from plasma as distinct from cell-derived origin. Therefore direct links of defining and validating EV and nonEV markers is dependent on their source material.

1A. We thank the reviewer for generally positive assessment of our analytical framework, and we appreciate raising this critical point. This comment highlights an area in our original text that clearly lacked sufficient emphasis, and we welcome the opportunity to unequivocally clarify our dataset composition.
To be absolutely clear: The core plasma EV meta-analysis dataset does not contain any cell-based EV studies. All 515 datasets analyzed in the primary deconvolution matrix are derived exclusively from human clinical plasma. We strongly agree with the reviewer's premise that plasma EVs are fundamentally distinct from cell-culture EVs, and that utilizing cell-culture EVs to define plasma markers is a flawed approach. It is precisely for this reason that our inclusion criteria for the meta-analysis strictly excluded in vitro cell-line studies.

We suspect the reviewer's (highly justified) concern stems from our use of cellular and tissue-level proteomic datasets (e.g., purified primary platelets, erythrocytes, and human liver tissue). We wish to clarify the distinct role of these studies:

1. *The Core Matrix: The 515 samples clustered in our analysis are strictly proteins from human plasma EVs.*
2. *The Reference Signatures: To accurately annotate the biological origin of the resulting plasma EV clusters, we compared those clusters against enriched primary cellular and tissue proteomes. For instance, to provide evidence that a specific module in the plasma EV isolate was actually a co-isolating platelet component, we referenced a pure platelet proteome.*

Therefore, our definition of EV vs. non-EV markers is entirely dependent on the in vivo plasma source material, exactly as the reviewer recommends.

*To improve clarity of the manuscript, we have revised the manuscript. We have explicitly stated in the Results section "*A Unified Proteomics Atlas of 515 Human Plasma EV Preparations.

To ensure biological specificity and avoid confounding signatures from other sources, our core dataset and all subsequent clustering analyses were restricted exclusively to human plasma EV datasets*" (Page 3). In addition, the corresponding sub title in method section was amended to "*Systematic Search and Selection of Public Plasma EV Proteomics Datasets".

R2 P2: One questions the use of the term contamination signatures. These should be removed given the nature of the sample matrix – you will never completely remove these, but rather focus on enrichment. For example complement proteins and immunoglobulins may bind EV surfaces, and Lipoproteins may physically associate with EVs. The key factor here is enrichment relative to the presence of these multiple biological module factors. This needs to be integrated throughout

AU. We completely agree with the reviewer. As noted in our prior responses, while our initial use of the term "contaminants" was drawn directly from the MISEV2024 guidelines (which classify these as "Category 3: matrix contaminants"), your critique perfectly captures why this binary term is biologically inadequate.
We have systematically removed the term "contamination" throughout the manuscript in favor of "co-isolates" and "plasma matrix". Furthermore, we have explicitly incorporated the reviewer's point regarding relative enrichment and the physical association of complement, immunoglobulins, and lipoproteins into the revised discussion section (Page 19).

R2 P3: Blood processing and plasma pre-analytical variation. Does the preparation, storage time of plasma impact the core proteome? These variables need to be introduced for the 20 studies profiled and built into the analysis framework.

AU: We strongly agree with the reviewer that pre-analytical variables (blood collection tubes, centrifugation steps, and storage time/temperature) are critical factors in plasma EV biology.
Ideally, we would have included these parameters as covariates in our clustering matrix. However, the reality of conducting a meta-analysis across historical datasets is the lack of standardized metadata reporting. Most of the 20 studies included in this analysis simply did not report exact biobanking durations, freeze-thaw cycles, or precise plasma preparation details in their supplementary materials. Because this data is overwhelmingly missing or unrecoverable, integrating it into the computational framework is unfortunately impossible without introducing severe imputation bias.

Nevertheless, we can address the reviewer's central biological question: Does storage time impact the core proteome? Recent biobanking studies have demonstrated that while long-term storage or freeze-thaw cycles may impact EV functionality, size distribution, or absolute particle yield, the fundamental structural proteome and the major co-fractionating complexes (such as lipoproteins) remain highly stable and identifiable (Witwer, K. W., Buzás, E. I., Bemis, L. T., Bora, A., Lässer, C., Lötvall, J., ... & Théry, C. (2013). Standardization of sample collection, isolation and analysis methods in extracellular vesicle research. Journal of Extracellular Vesicles, 2(1), 20360.; J Extracell Vesicles. 2024 Feb;13(2):e12404. doi: 10.1002/jev2.12404. Erratum in: J Extracell Vesicles. 2024 May;13(5):e12451. doi: 10.1002/jev2.12451. PMID: 38326288; PMCID: PMC10850029).

In our global co-variance analysis, the clustering of co-isolate signatures captures variance driven by a combination of isolation methods, MS platforms, and preanalytical variables such as storage time. However, by breaking down the apparent abundance of co-isolates per method, our data demonstrates that this massive co-isolate burden is a universal bottleneck across all techniques. This overarching methodological

limitation effectively overshadows the subtler technical noise introduced by individual preanalytical differences.

To ensure this critical limitation is transparent to the reader, we have added a dedicated paragraph to the Discussion (Page 19). This new section explicitly acknowledges the unmeasured impact of pre-analytical variables, cites the relevant literature on core proteome stability, and reinforces the urgent need for comprehensive metadata reporting (MISEV guidelines) in future proteomics studies.

R2 P4: Consistency. In the study 344 proteins are reported across studies at identification rate >50%. When this is increased to 80%, do these key markers still remain dependent on the type of approach to isolate and proteomically analyse? Why 50%? Isn't this just a proteomic dynamic range factor rather than the core composition of an EV? Is this the same peptides being identified across different studies that comprise this core atlas? How was the data overall normalised or was this a single batch analysis framework? How were batch corrections and random effects modelling performed? This needs to be rigorously built in for the formal analysis and figures.

AU: We thank the reviewer for these rigorous methodological queries. These concerns align very closely with those raised by Reviewer 1. We completely agree that the robustness of the core threshold and the handling of inter-study variance are paramount. We address these points as follows, and have implemented significant updates to our formal analysis to address them:

1. The 50% Threshold, Dynamic Range, and Sensitivity Analysis: We selected the 50% threshold specifically to account for the stochastic nature of historical label-free Data-Dependent Acquisition (DDA) mass spectrometry. In highly complex DDA matrices, even universally present core proteins will inevitably suffer from stochastic missing values (often dropping below an 80% identification rate simply due to MS1 peak-picking limitations, independent of biology). Pushing the threshold to >80% without justification would therefore artificially truncate the core plasma EV proteome due to instrument mechanics.

However, to definitively demonstrate that our biological modules are robust and not an artifact of this specific 50% threshold, we have performed a comprehensive sensitivity analysis evaluating cutoffs of 30%, 50%, 70%, and 90%, exactly as requested by Reviewer 1. This analysis confirms two critical points: First, the fundamental architecture of the biological modules, and their dependency on specific isolation approaches, remains highly conserved across all cutoffs. Second, altering the threshold does not change our overarching semi-quantitative conclusion, the vesicular signature consistently appears at a lower abundance than the co-isolate abundance, regardless of how strictly the data is filtered. This formal analysis has been added to the revised manuscript (Fig. S4).

2. Uniform Raw Data Re-Processing and Peptide/Protein Aggregation: We realize that we did not emphasize a critical methodological strength of this study sufficiently in the original text (Page 21, section "Unified Raw Data Processing (MaxQuant Workflow)"). We did not rely on the heterogeneous protein identification lists published in the original studies. Instead, we retrieved the raw mass spectrometry data files for all 515 isolates and performed a massive, uniform re-analysis from scratch using a single, standardized MaxQuant pipeline. Therefore, the peptide identification engine, mass tolerance, FDR thresholds (strictly 1% at both peptide and protein levels), and protein grouping algorithms were mathematically identical across the entire meta-analysis.

3. Normalization and Batch Effects: As detailed in our response to Reviewer 1, applying traditional variance-removal batch correction algorithms (such as ComBat) to this specific dataset would be mathematically counterproductive. Because different studies used fundamentally different physical isolation methods (SEC, UC, Precipitation), the batch effect between studies is inextricably linked to the physical co-

isolation of distinct biological networks (the functional matrix). Formal batch correction would mathematically force an SEC isolate to look identical to a precipitation isolate, erasing the very biases we aimed to measure. Instead, to neutralize technical variance (e.g., differing MS instrument sensitivities and sample loading amounts) while preserving the physical method variance, we utilized non-parametric rank normalization.

R2 P5: The composition of plasma EVs. While significant focus on the plasma EV itself, what about other co-isolated protein modules. Can these be compared to large scale plasma proteome efforts? In context of Rai et al as described, can the non-EV components identified consistent in this study from plasma also be contrasted here. Are these just contributed by the protein corona where knowledge in this at a proteome level interacting with various nanoparticles is being presented, eg Blume et al 2020.

AU: We completely agree with the reviewer's points. To rigorously address these, we have conducted formal comparative analyses and added entirely new Results sections explicitly benchmarking our core modules against large-scale reference datasets for whole plasma abundance, synthetic nanoparticle coronas (Blume et al., 2020), and agonist-induced platelet secretomes (Moon et al.). We have also significantly expanded our Discussion to contextualize these findings:

1. Comparison to the Whole Plasma Proteome: If EV isolation merely captured random plasma background, the resulting non-EV signals should perfectly mirror whole plasma abundance (which is massively dominated by Albumin and a broad smear of unspecific immunoglobulins). Instead, as detailed in our new plasma abundance mapping Results section, our deconvolution reveals highly structured, distinct biological networks (e.g., specific high-density lipoprotein signatures, defined complement cascades, and targeted hepatic acute-phase networks). This strongly indicates that while the recovery of these proteins is heavily influenced by a concentration-driven sampling bias inherent to the plasma matrix, they do not represent random carryover. Rather, they are specifically co-fractionating entities driven by this mass-action combined with shared biophysical characteristics.

2. The Biomolecular Corona (Blume et al.): The reviewer's insight regarding the biomolecular corona is perfectly aligned with our findings. To formally test this, we systematically cross-referenced our core dataset with the comprehensive biomolecular corona profiles of 11 distinct synthetic nanoparticle chemistries reported by Blume et al. As presented in the updated Results, this comparative analysis confirms that a substantial fraction of the co-isolated soluble modules functionally behaves as an electrostatic plasma corona, actively adsorbing to vesicular surfaces rather than existing as purely independent background noise.

3. Hematopoietic and Platelet Contributions (Moon et al.): To investigate the cellular origin of the vesicular fraction, we benchmarked our core proteome against agonist-induced platelet release profiles from Moon et al. As detailed in the new Results section, we observed a significant correlation between the plasma EV core and the platelet secretome. Notably, while significant, this correlation was lower in magnitude than the correlation observed with the Blume nanoparticle corona data, further reinforcing our conclusion that the bulk plasma EV isolate is heavily dominated by the soluble electrostatic matrix. However, the platelet-derived fraction remains the defining feature of the actual vesicular compartment; as demonstrated by our subsequent co-variance clustering analysis, canonical EV signals (e.g., CD9, ADAM10) are inextricably linked to this hematopoietic shedding network, physically distinguishing them from generic tissue exosomes.

4. Consistency with Rai et al.: We have added text explicitly contrasting our modules with the foundational work of Rai et al. As Rai et al. elegantly demonstrated, classical EV markers often co-elute with major non-vesicular plasma components. Our 515-sample meta-analysis strongly validates and scales Rai's

observations, providing a mathematical architecture that maps exactly which non-vesicular components described by Rai consistently form distinct physical modules across different isolation techniques.

We are grateful to the reviewer for highlighting these specific studies, as integrating the concepts of Moon et al. and the biomolecular corona of Blume et al. has fundamentally elevated the conceptual framework of our Discussion.

R2 P6: A key finding is identification of CD9, CD81, and ADAM10 cluster with platelet and erythrocyte proteins, suggesting that their signal in plasma largely derives from hematopoietic ectosomes rather than generic EVs. Can other studies be included here to support such statements. What about platelet activation markers or RBC shedding markers from proteomics across the same 20 studies? What about platelet activation EV release factors at a proteome level (e. Moon 2024). Co-variation reflects shared cellular origin, not misannotation. This wording and the way in which this deconvolution is being performed needs to be improved for accuracy and clarity.

AU: We deeply appreciate this comment, particularly the reviewer's concluding point: "Co-variation reflects shared cellular origin, not misannotation". This perfectly encapsulates the biological reality of plasma EVs, and we agree.

As noted in our responses to prior comments, this conceptual shift is exactly why we have systematically removed the terminology of "contaminants" throughout the revised manuscript. When classical markers like CD9 and CD81 strongly co-vary with platelet and circulating lymphocytes networks, it indicates a shared hematopoietic origin, not a technical artifact.

We strongly agree with the reviewer's fundamental perspective: co-variation in this context reflects a shared cellular origin, not "misannotation". Hematopoietic vesicles are bona fide EVs, and our goal is to accurately define their dominant cellular lineages in the complex matrix of bulk plasma. We thank the reviewer for pointing out that our previous wording lacked this clarity.

Our mathematical analysis grouped canonical markers CD9, ADAM10, and CD81 into a single co-varying module (Cluster 7). To biologically deconvolute the specific cellular drivers within this shared network, we compared our cluster against deep-proteomic profiles of agonist-induced platelet EVs (Moon et al., 2024), utilizing whole-platelet proteomics strictly for additional cellular context:

1. *CD9 and ADAM10 (The Platelet Driver): Both of these canonical markers rank among the absolute most abundant proteins in the platelet EV preparations reported by Moon et al. This direct EV-to-EV comparison definitively suggests that bulk platelet shedding is the massive driver of the CD9 and ADAM10 signals within this cluster.*
2. *CD81 (The Lymphocyte Driver): Interestingly, while CD81 co-varies in this same cluster, it is notably absent from the Moon platelet EV dataset. It is well-established in foundational cellular immunology that CD81 in the blood is heavily derived from lymphocytes, serving as a critical surface protein on both B cells and T cells. The presence of CD81 in Cluster 7 therefore highlights the composite nature of this network: it mathematically captures the biophysical co-isolation of platelet EVs alongside lymphocyte EVs.*

Manuscript Updates for Accuracy and Clarity: Guided by the reviewer's feedback, we have thoroughly revised the text to remove any implication of "misannotation". We have also renamed Cluster 7 from "mainly platelet EVs" to the more accurate "Hematopoietic-Derived EV Network" (Page 18 and Figure 7&8). Furthermore, we have added text to the Discussion utilizing the Moon et al. data to deconvolute the distinct platelet and lymphocyte origins of the canonical markers within this single composite cluster.

R2 P7: Check spelling and details in figures and throughout. E.g., figure 4-5; platlets

AU: Thanks now corrected.

R2 P8. What was the inclusion criteria for these 515 proteomes? It seems just a search parameter focus was used – and that the detail in the stringency of obtaining an EV was not given? Some are poor quality based on coverage/depth, and correlation with their EV. A clear focus on why these 515 (and 20 studies) specifically is needed. Why not focus just on the 50% of study proteomes that are being used to generate the core and comparative elements of the study? Are the remaining 50% EV at all, or just plasma proteome sets? This detail need careful focus, given the narrative is for the analysis and interpretation of EVs.

AU: We thank the reviewer for highlighting the need for absolute clarity regarding our cohort selection. The reviewer's comment indicates that our original description of the data filtering led to a critical misunderstanding, which we are eager to correct.

1. Clarification of the 50% Threshold: The 50% threshold did not apply to the exclusion of samples or studies. All 515 isolates from all 20 studies were retained and analyzed. The 50% threshold was applied strictly to protein detection frequency across the cohort (i.e., a specific protein had to be detected in at least 50% of the 515 runs to be evaluated as a highly reproducible core component). Therefore, there are no "remaining 50% of study proteomes" that were discarded; the entire 515-sample matrix was utilized to map the comparative elements of the study.

2. Explicit Inclusion/Exclusion Criteria: We have now added a dedicated "Systematic Inclusion/Exclusion Criteria" subsection to the Methods. In brief, datasets were exclusively selected if they met all of the following strict criteria:

- *Derived from human plasma or serum.*
- *Published in peer-reviewed journals with the explicit author-defined claim of isolating EVs.*
- *Analyzed via Label-Free, Gel-Free, Data-Dependent Acquisition (DDA) LC-MS (to ensure uniform downstream analytical mechanics).*
- *Raw data publicly available for uniform MaxQuant reprocessing.*

This core dataset of 515 runs was further supported by the targeted inclusion of DIA datasets that was used for orthogonal validation.

3. The Rationale Regarding "Stringency" and "Dataset Depth": The reviewer notes that some included datasets might be considered "poor quality based on coverage/depth" or lack high stringency. This observation strikes at the fundamental premise of our meta-analysis. Our objective was not to artificially curate a "perfect" subset of highly stringent EV studies. Rather, our goal was to perform an unbiased, real-world evaluation of what the global field currently produces and publishes under the umbrella term of plasma EVs. If we had preemptively excluded studies based on subjective assessments of "low stringency", we would have introduced massive selection bias and erased our ability to mathematically map the co-isolating functional networks (or corona) that plague standard methodologies. Furthermore, in plasma EV proteomics, raw MS depth (total protein count) is often inversely correlated with EV purity; a highly stringent physical isolation often yields a shallow dataset because the thousands of abundant background plasma proteins have been successfully depleted. By applying non-parametric rank normalization to the uniform MaxQuant output, our pipeline successfully extracted the biological modularity from both shallow and deep datasets without bias.

We believe that outlining these explicit criteria and our rationale for real-world inclusivity fundamentally strengthens the transparency of the manuscript.

R2 P8: The exclusion of DIA centric data is a major limitation. The expansion in DIA with EV proteome studies needs to be factored in. Not a written explanation but integration of various DIA-human EV plasma sets to validate the core EV elements and other findings. The use of DIA is more prevalent in the past 2-3 years, and has overcome significant limitations in acquisition, coverage and quantitative accuracy.

AU: We strongly agree with the reviewer. DIA is undeniably the current gold standard for quantitative proteomics, and validating our DDA-derived architecture in modern DIA datasets is a brilliant recommendation that fundamentally elevates the manuscript.
Our initial exclusion of DIA data from the primary 515-sample matrix was a deliberate mathematical necessity. Because DDA and DIA possess fundamentally different architectures for missing values (stochastic MNAR vs. continuous sampling), integrating them into a single clustering algorithm would have introduced a severe analytical batch effect, artificially dividing the clusters by MS acquisition mode rather than biological co-isolation.

However, we completely agree with the reviewer's suggestion to use DIA datasets to validate the core findings. To achieve this, we have curated an independent DIA Validation Cohort.

1. *The DIA Analysis: We aggregated 2 recent, high-quality DIA human plasma EV studies (Suresh et al and Wu et al).*
2. *The Validation: We applied the new co-isolation burden independently to this DIA cohort.*
3. *The Results: As the reviewer predicted, the superior quantitative accuracy of DIA robustly validated our core findings. The DIA matrix successfully resolved the same core vesicular machinery and the major co-isolating functional networks (e.g., lipoproteins, immune complexes, and platelet networks).*

By treating the DIA data as an orthogonal validation cohort, we demonstrate that the biological modularity of plasma EVs we describe is a universal physical phenomenon, not an artifact of DDA acquisition.

We have added a entirely new sub-section to the Results (Page 9 and 10), alongside a new Main Figure (Fig. S6) detailing this DIA integration.

Reviewer 3:

R3 P1:The distinction between EVs and contaminants, found in almost all purification strategies, is a critical issue in the EV field. This is all the more true for complex biological fluids such as blood plasma, which is an important source of biomarkers. Performing a meta-analysis from a large set of proteomic studies, the authors first identify a reproducible core proteome of 350 proteins based on their frequency of detection (>50%) in the LC-MS runs, and then, characterize the content of this core proteome by clustering strategies. They demonstrate that this core proteome mainly consists in contaminating non-EV proteins but further refine its composition into 8 biological modules, thus replacing the binary EV non-EV subdivision. An important finding is the clustering of ectosome (plasma membrane signature) markers, but not exosome markers (endosomal signature), with the hematopoietic lineage, which is highly relevant when searching for biomarkers. I will not comment on the bioinformatic tools used in this study because of lack of expertise but restrict my comments to the global strategy and the conclusions drawn by the authors.

AU: We sincerely thank Reviewer 3 for the time and expert evaluation of our global strategy, and this highly accurate and encouraging summary of our work.

R3 P2: It is classically reported that the nature of contaminants mainly depends on the purification strategy, as shown also in sup Fig2. Almost 60% of the individual runs analysed in this study derived from only 2 different purification strategies (fig1a), and 20% likely issue from a single lab (RNeasy purification). It

challenges the definition of the core proteome which is based on the frequency of detection of proteins in all individuals MS-runs without weighting the data. For instance, the detection frequency of the core proteins relative to the number of studies gives a different picture (Fig3b), often being under the 50% threshold. How the different clusters identified by the authors distribute depending on the purification strategy might be informative for the selection of the most appropriate one.

AU: This is a very good point. We have now provided an additional plot dividing the data into isolation methods. We initially were hesitant in performing this analysis since preanalytics, MS sample preparation and MS instrumentation additionally will affect the results and are not available information to be included as co-variates in method comparison. Nevertheless, the actual analysis reveals overall similar structure using our co-isolation burden based analysis (Fig. S5).

R3 P3: The authors nicely confirm the abundance of non-EV contaminants in EV preps (although now widely accepted in the field) but might more deeply comment their findings with those of other previous studies (e.g. Vallejo 2023).

AU: We appreciate this excellent suggestion and completely agree that our results should be explicitly contextualized within the broader landscape of recent meta-analyses. We have expanded our Discussion (Page 17) to integrate our findings with these large-scale evaluations, explicitly citing the foundational work by Vallejo et al. (2023).
In the revised text, we note that while previous meta-analyses have firmly established the widespread presence of non-EV proteins across standard isolation methods, our analysis builds directly upon this consensus. Specifically, we demonstrate that these widely accepted co-isolates do not merely represent stochastic background noise, but rather resolve into highly reproducible, co-fractionating biological modules (such as the lipoprotein matrix and the hematopoietic-derived EV network). We thank the reviewer for highlighting this study, as it provided the perfect foundation to frame our core modularity concept.

R3 P4: From the literature, tissue-derived EVs represent at maximum few % of plasma EVs. The fact that "the EV proteome signal in bulk plasma is primarily driven by the burden of hematopoietic ectosomes rather than generic tissue-derived exosome secretion" is thus not unexpected. This has to be quoted . Focusing on the core proteome might hinder the detection of this minor component of the EV proteome. From the authors findings, are alternative and more directed bioinformatic strategies could be consider to target specifically this population?

AU: We strongly agree with the reviewer's perspective. Our mathematical findings perfectly align with foundational flow cytometry and single-vesicle literature, which have long estimated that the vast majority of circulating EVs are derived from platelets and erythrocytes, while tissue-derived EVs represent a minute fraction of the total plasma pool.

1. Contextualizing with the Literature: As requested, we have added explicit text and citations to the Discussion (Page 18 and 19) to formally acknowledge this. We note that our findings are not unexpected in this context, but rather serve as the definitive proteomics confirmation of this physiological reality. We have cited foundational work such as Arraud et al., 2014, and recent clinical flow cytometry studies confirming tissue EVs are a rare minority).

2. Acknowledging the Limitation of the Core: We completely agree that by focusing on proteins detected in >50% of runs, our algorithm acts as a high-pass filter. The core proteome we defined is fundamentally a map of the dominant, ubiquitous background (the hematopoietic EVs and the co-fractionating functional matrix). By definition, this approach actively filters out and hinders the detection of rare, tissue-specific EV biomarkers (which might only be present in 5% of disease-specific clinical samples). We have explicitly stated this limitation in the revised Discussion (Page 20, top).

3. Alternative Bioinformatic Strategies: Based on our findings, we propose two alternative bioinformatic strategies, which we have now detailed in the concluding paragraph of the manuscript (Page 20):

Strategy A: In Silico Background Subtraction: The eight ubiquitous modules defined in this study (e.g., Platelet, Erythrocyte, Lipoprotein, and Complement clusters) provide a standardized bioinformatic exclusion list. By computationally subtracting the Mass Spectrometry (MS) signal of these dominant background modules from any new clinical dataset, we can substantially increase analytical sensitivity, revealing underlying, low-abundance tissue-specific EV signatures.

Strategy B: Supervised Deconvolution Architectures: Our study utilized unsupervised clustering, which naturally resolves only the most abundant signals. For future pipelines, we propose transitioning to supervised bioinformatic deconvolution (analogous to CIBERSORT algorithms used in bulk tissue transcriptomics). By training machine-learning algorithms on pure, isolated tissue-EV signatures (e.g., brain, liver, or tumor-derived EVs), it will be possible to computationally extract and quantify the fractional contribution of these minor populations from complex bulk plasma EV signals.

Reviewers' comments:

Reviewer #1 (Remarks to the Author):

R1: I would like to thank the authors for the detailed and rigorous answers for each point of my previous reviewing. This reviewing step has been led in a state-of-the-art manner and answered to all my questions or remarks, and the quality of the manuscript is now indiscutable.

A: We thank reviewer 1 for time invested and useful comments.

Reviewer #2 (Remarks to the Author):

All key areas and queries raised have been addressed and content in the revised manuscript updated. well done and happy to proceed here

A: We thank reviewer 2 for time invested and useful comments.

Reviewer #3 (Remarks to the Author):

R: In this revised version of the manuscript, the authors provide a valuable set of new analyses, including the ones I suggested. May be the most instructive is the inclusion of DIA data which appear to corroborate the DDA analysis and be more robust across purification methods (figure S6, abbreviation of each method should be defined, e.g. TEI), also reflecting the nature of the starting material (Wu et al study). May be the DDA/DIA comparison might be further comment in the discussion and one figure included in the main text. The inclusion of the corona composition based on Blume et al. study also brings some new insights although reducing EV binding properties of corona proteins just to their pI looks rather simplistic and could be nuanced in the discussion. Beyond these suggestions. The new data and answers provided by the authors justified the acceptance of the manuscript.

A: We thank Reviewer 3 for the time invested and the highly useful comments. We have now added the definitions for the abbreviations TEI and NPR in the Figure S6 legend. We thank the reviewer for highlighting the value of the DDA/DIA comparison, and we entirely agree that this is a robust and instructive analysis. While we have expanded our commentary regarding this comparison in the main text Discussion as suggested, we have opted to retain the visual data as Supplementary Figure S6. The main manuscript currently contains eight dense, multi-panel figures, and we felt that introducing a ninth would exceed ideal formatting constraints and disrupt the primary narrative flow. Finally, we have added further nuance to the main text Discussion acknowledging that EV binding properties and corona formation are multifactorial, extending beyond just the isoelectric point (pI), as astutely suggested.
