# Supplementary material for: Analysis of 515 human proteomes defines plasma extracellular vesicle core composition, corona and coisolated networks
Source: Commun Biol. 2026 Oct 30;9:1402. doi: 10.1038/s42003-026-11038-6 (PMC13722686; doi:10.1038/s42003-026-11038-6)
Supplement: Supplementary file 2 [file 42003_2026_11038_MOESM2_ESM.pdf]

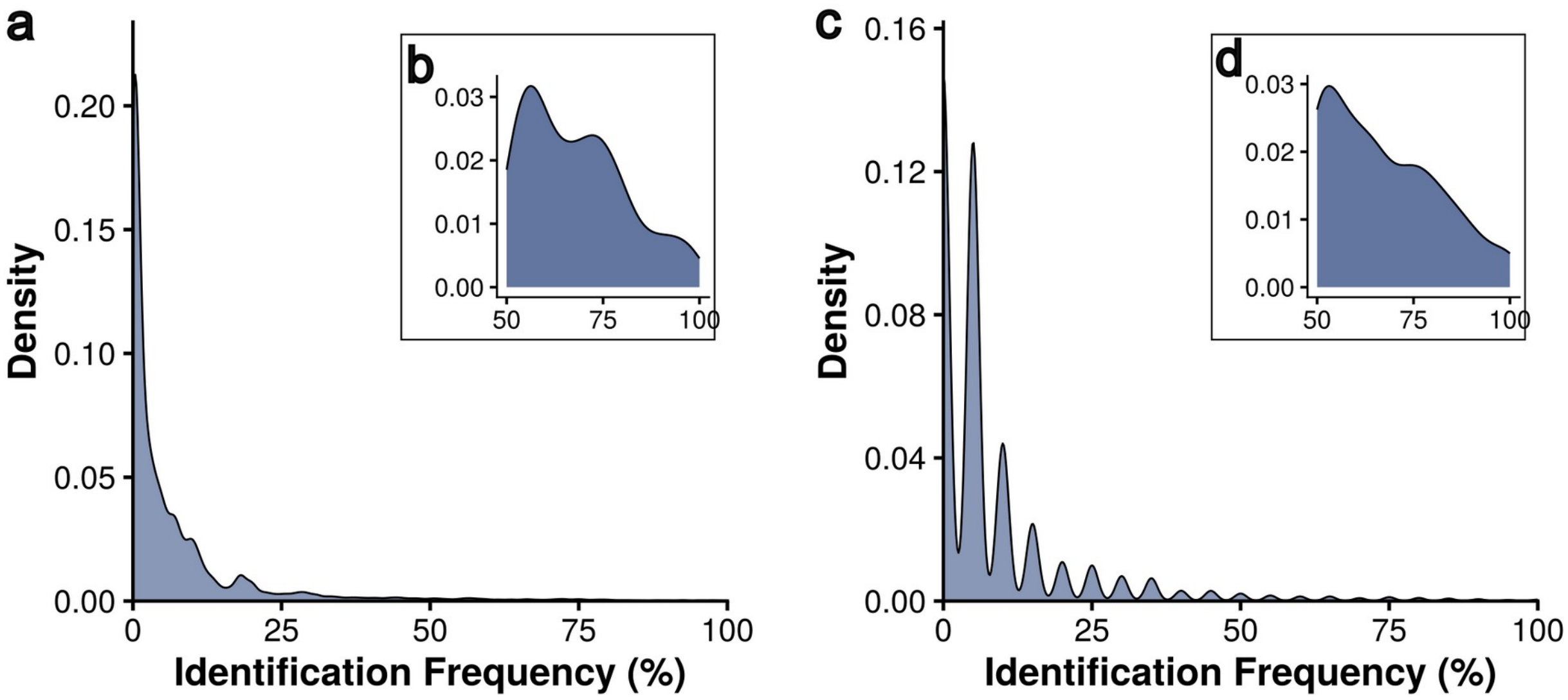

Fig. S1 **|** *Identification frequency across* ***(a** & **b)*** *LC-MS runs and* ***(c** & **d)*** *studies.*

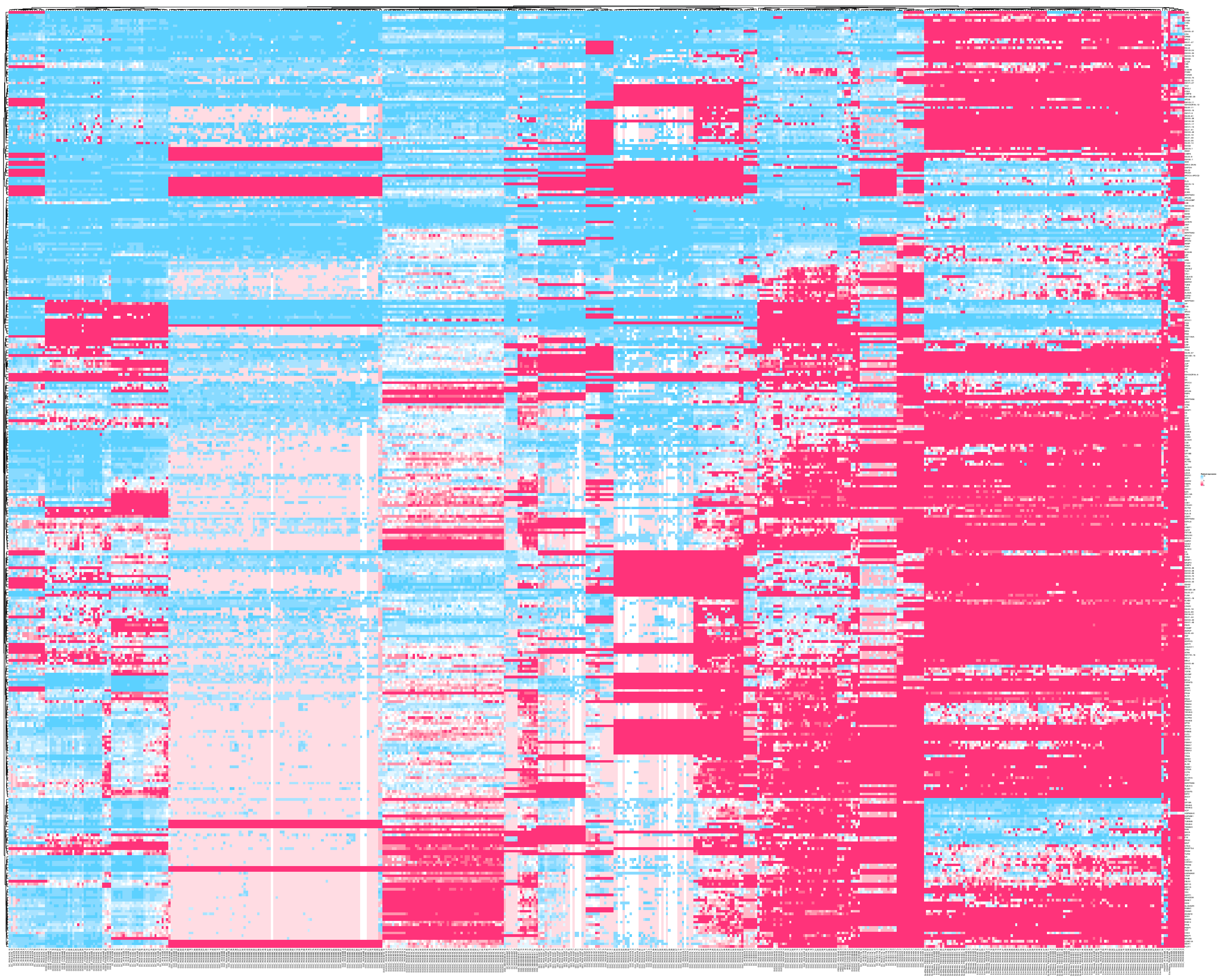

Fig. S2 | Heatmap with rank normalize protein expression values of proteins identified in more than 50% of all LC-MS runs.

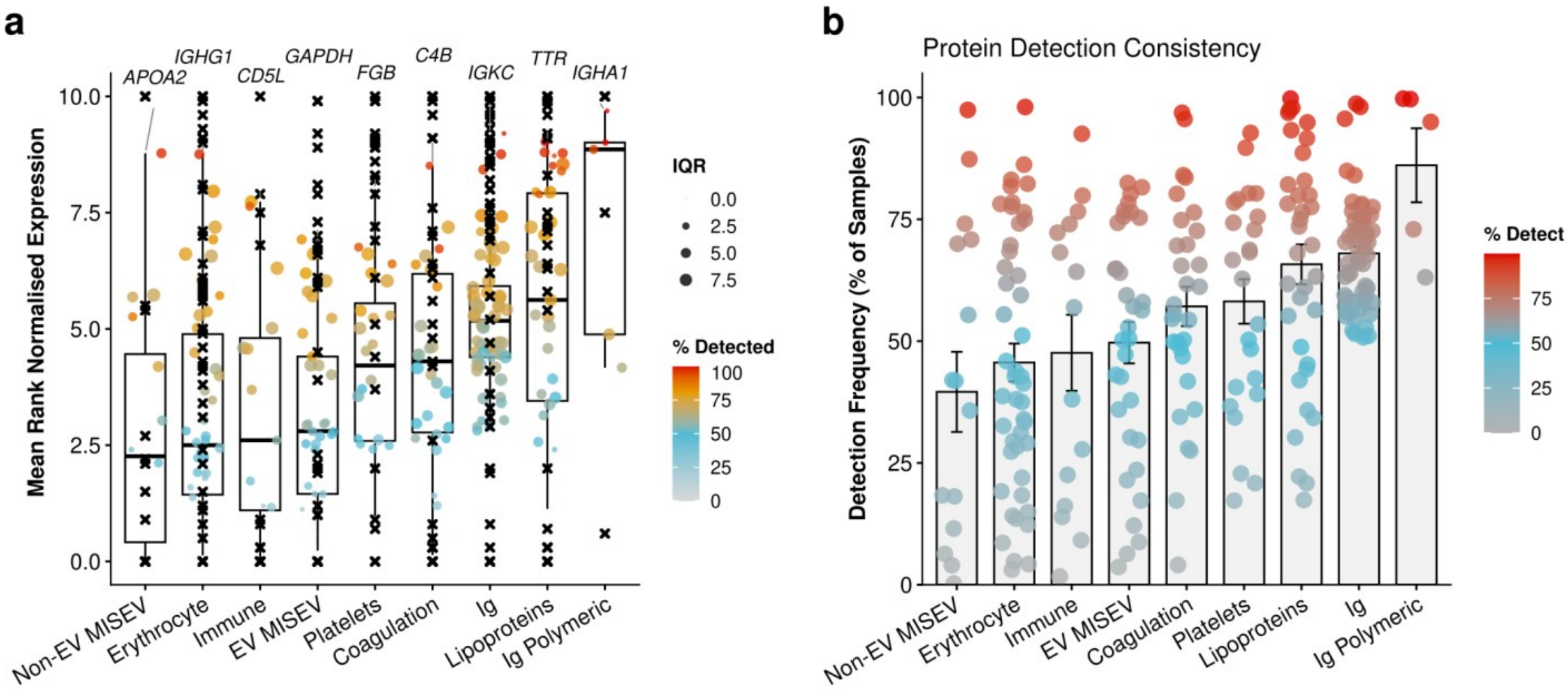

***Fig. S3 | Quantitative abundance and detection consistency of core EV markers in comparison with less strict non-EV markers signatures. a** Boxplots displaying the mean rank normalized iBAQ abundance of established MISEV EV markers (e.g., CD9, CD81) compared to common non-EV co-isolates (Lipoproteins, Erythrocyte proteins) across the entire dataset. The gene labels above each category are indicating the most abundant protein in the core EV plasma proteome for the respective category. The central line represents the median, box limits indicate the 25th and 75th percentiles, and whiskers extend to 1.5× IQR. The crosses indicate abundance values from density gradient isolation of plasma EVs. **b** Detection frequency of each protein category, calculated as the percentage of the 515 LC-MS runs in which the marker was identified. Note that non-EV proteins are both abundant (**a**) and exhibit high detection consistency compared to core EV markers (**b**). To avoid artificial variance suppression from zero-imputation ties, the Interquartile Range (IQR) of rank-normalized values across samples was calculated strictly across non-zero detection events.*

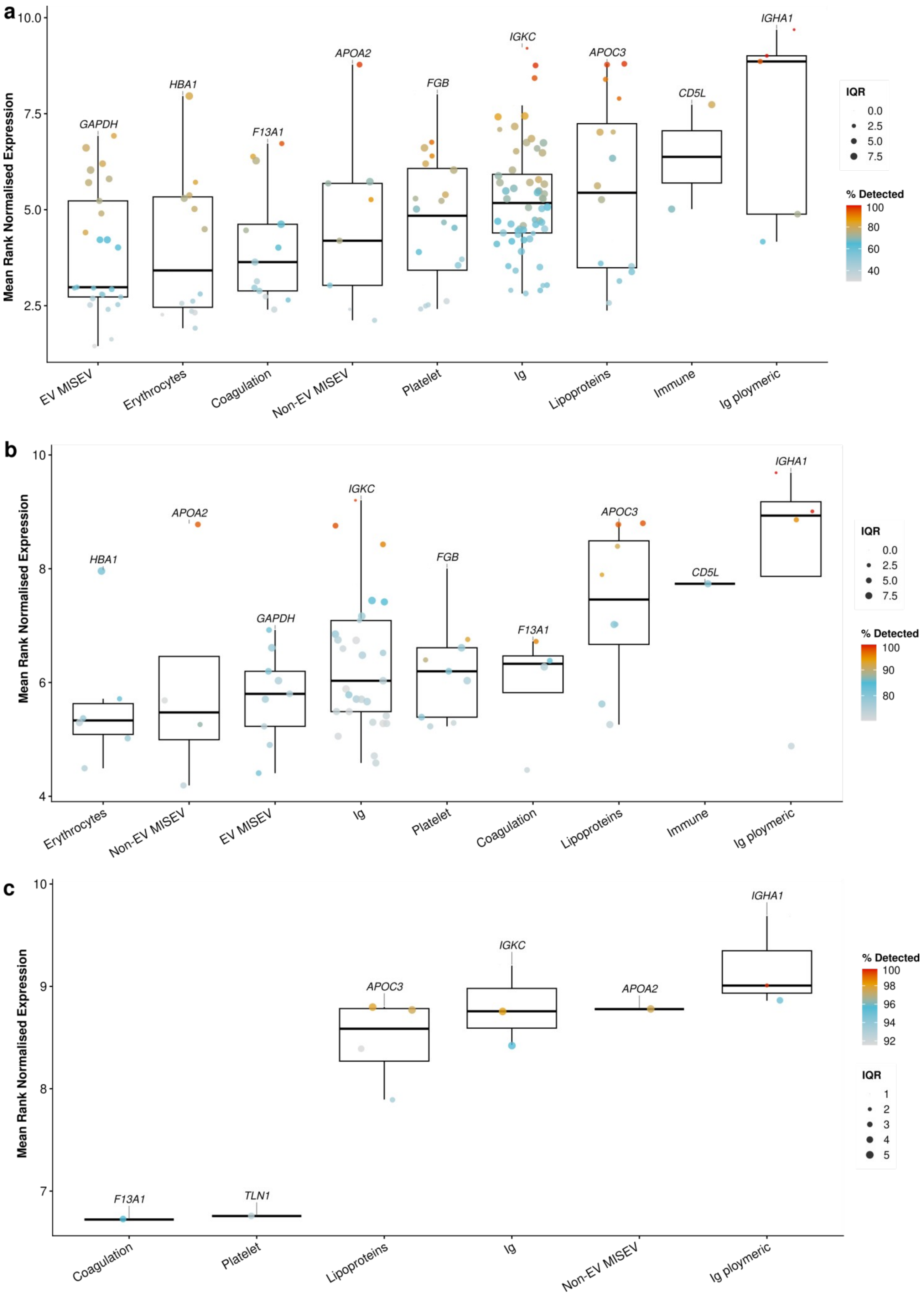

Fig. S4 | Sensitivity analysis of plasma EV co-isolate abundance across progressive detection frequency thresholds. *To ensure that the dominant abundance of non-EV proteins was not driven by*

sparsely detected artifacts across the 515-LC-MS meta-analysis, a sensitivity analysis was performed applying increasingly strict detection frequency thresholds. Boxplots display the mean Log_{10} *expression of plasma core EV markers and co-isolate signatures for proteins detected in at least* ***(a)*** *30%, (****b****) 70%, and (****c****) 90% of all included studies. The dot size corresponds to the coefficient of variation (CV), and the color gradient represents the specific percentage of studies in which the protein was detected. As detection stringency increases from 30% to 70%, the disparity in abundance between MISEV EV markers and major plasma co-isolates (e.g., Lipoproteins, Polymeric Ig) widens. Strikingly, at the highly stringent 90% detection threshold (****c****), canonical EV markers (EV MISEV2018) fail to meet the criteria and are entirely eliminated from the analysis, whereas plasma co-isolates remain consistently detected at high abundance.*

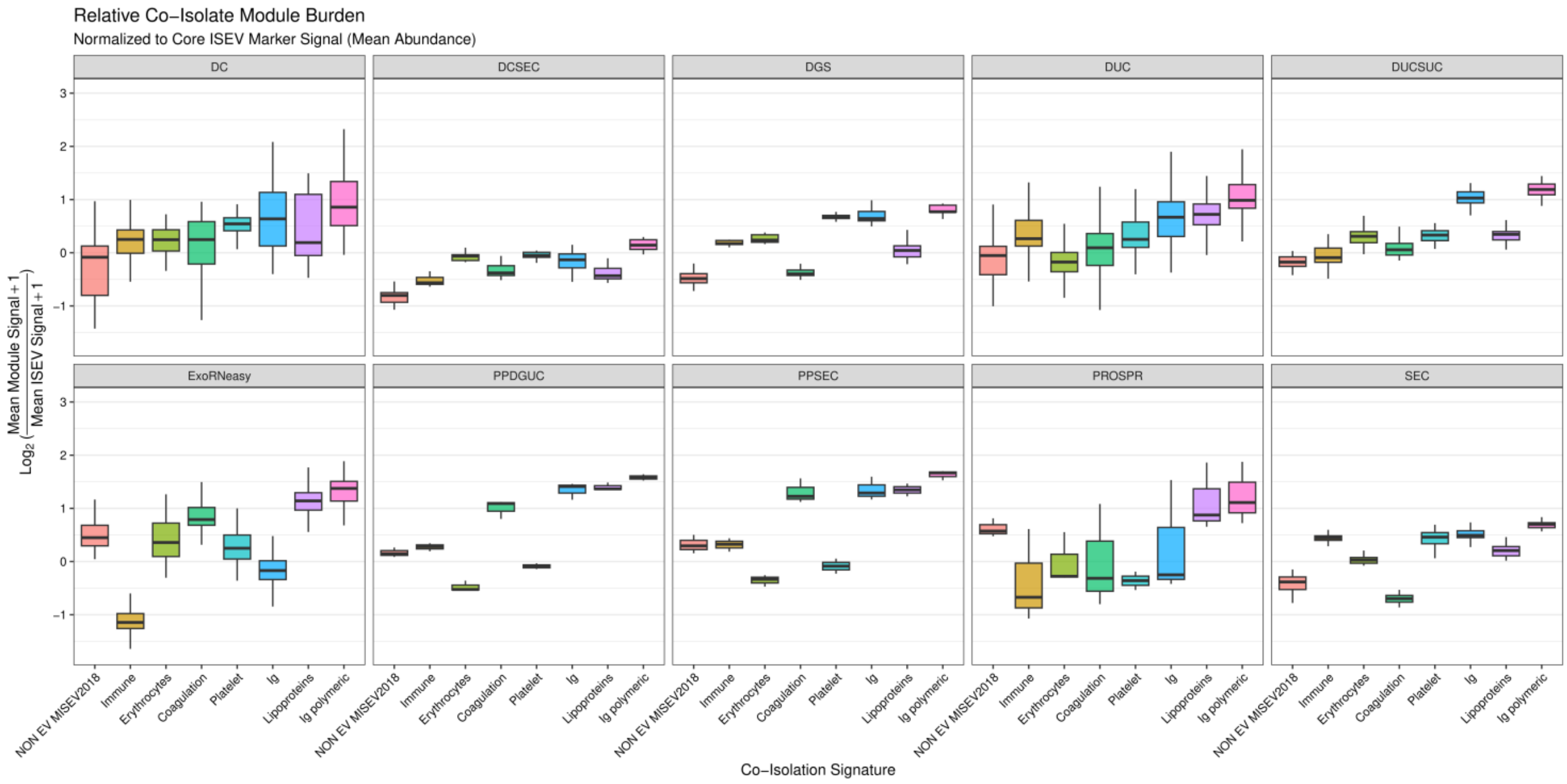

Fig. S5 | Relative co-isolate module burden stratified by physical EV isolation methodology (DDA datasets). *Boxplots depict the relative abundance of distinct plasma co-isolation signatures compared to an internal core EV marker baseline across the 515 data-dependent acquisition (DDA) datasets, faceted by specific EV isolation techniques. The burden metric is calculated as the log*$_2$*-transformed ratio of the mean quantitative signal of the designated co-isolate module to the mean signal of core MISEV structural markers (pseudocount +1). A score greater than zero indicates that the cumulative abundance of the co-isolate module exceeds the abundance of MISEV EV structural proteins. While minor quantitative variations exist between different physical isolation methods (e.g., differential ultracentrifugation [DUC], size exclusion chromatography [SEC], and polymer-based precipitation [PP]), a substantial co-isolate burden remains a ubiquitous phenomenon.*

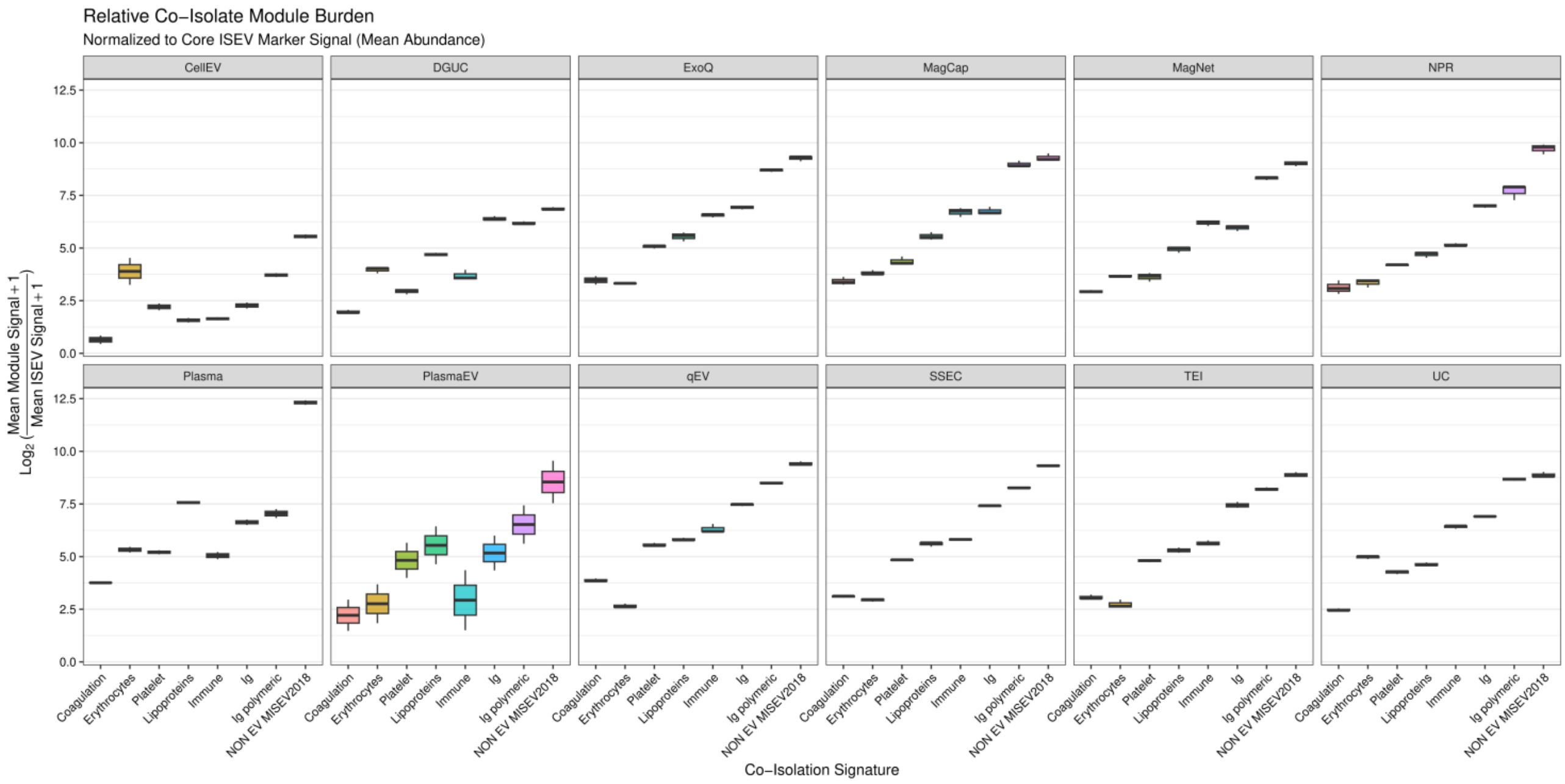

Fig. S6 | Orthogonal validation of relative co-isolate module burden across diverse EV isolation methodologies using Data-Independent Acquisition (DIA) mass spectrometry. *Boxplots depict the relative abundance of plasma co-isolation signatures in EV preparations analyzed via DIA (data derived from Suresh et al.). The burden metric is calculated as the* log_2*-transformed ratio of the mean quantitative signal of the designated co-isolate module to the mean signal of core MISEV structural markers (pseudocount +1). Data are stratified by the array of distinct physical and commercial EV isolation techniques evaluated in the original study (e.g., density gradient ultracentrifugation [DGUC], size exclusion chromatography [SSEC], and polymer precipitation [Norgen's Purification Resin (NPR), total exosome isolation (TEI)]).*

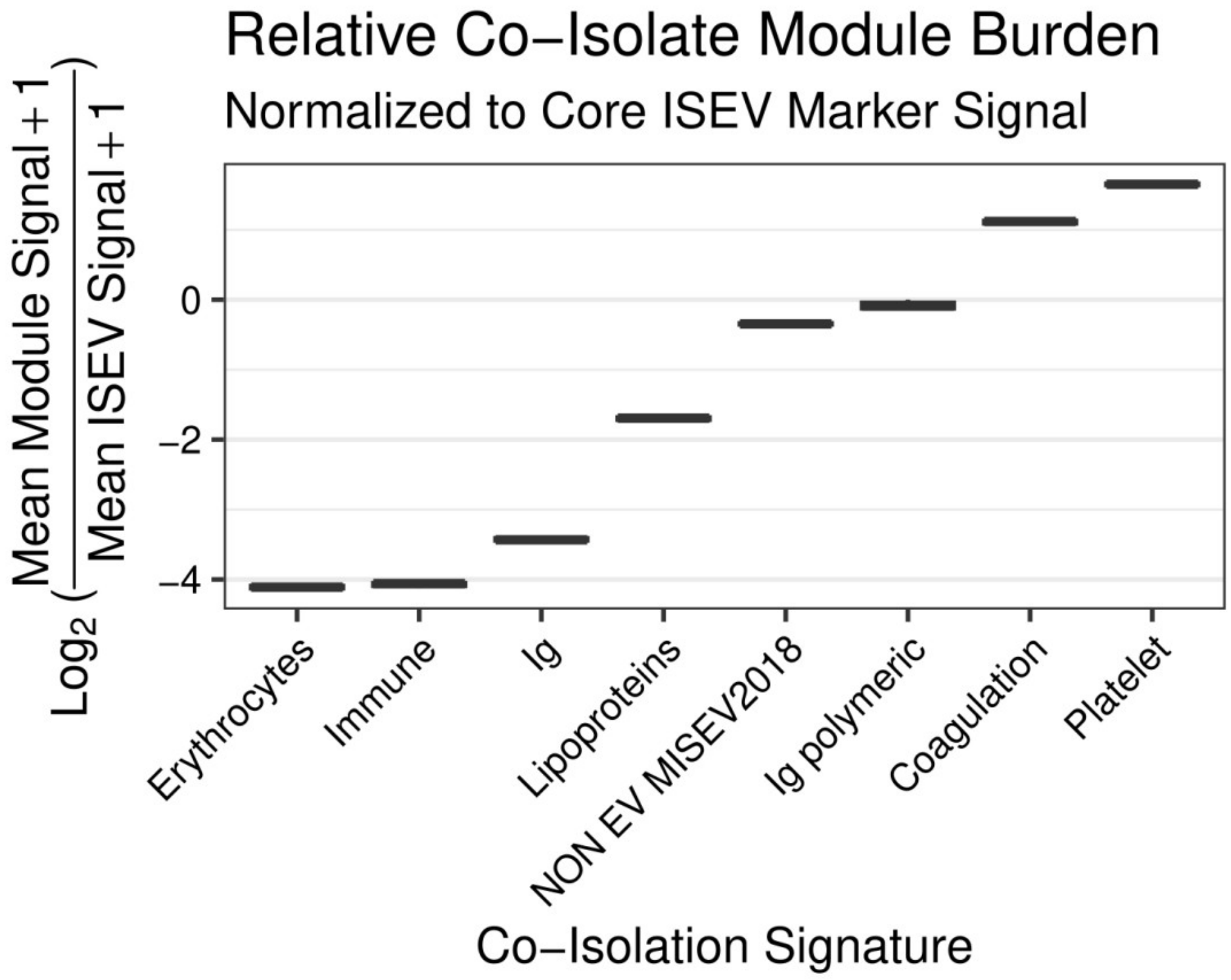

Fig. S7 | Influence of source biofluid composition on relative co-isolate module burden (Wu et al. DIA dataset). *The boxplot illustrates the relative abundance of distinct co-isolation signatures compared to an internal core EV marker baseline (log$_2$-transformed ratio with a pseudocount of +1) using Data-Independent Acquisition (DIA) mass spectrometry. In stark contrast to datasets utilizing standard platelet-depleted plasma, the platelet module exhibits the highest relative burden. This distinct profile directly reflects the specific methodological choice in Wu et al. to utilize platelet-enriched plasma as the starting biofluid for EV isolation.*

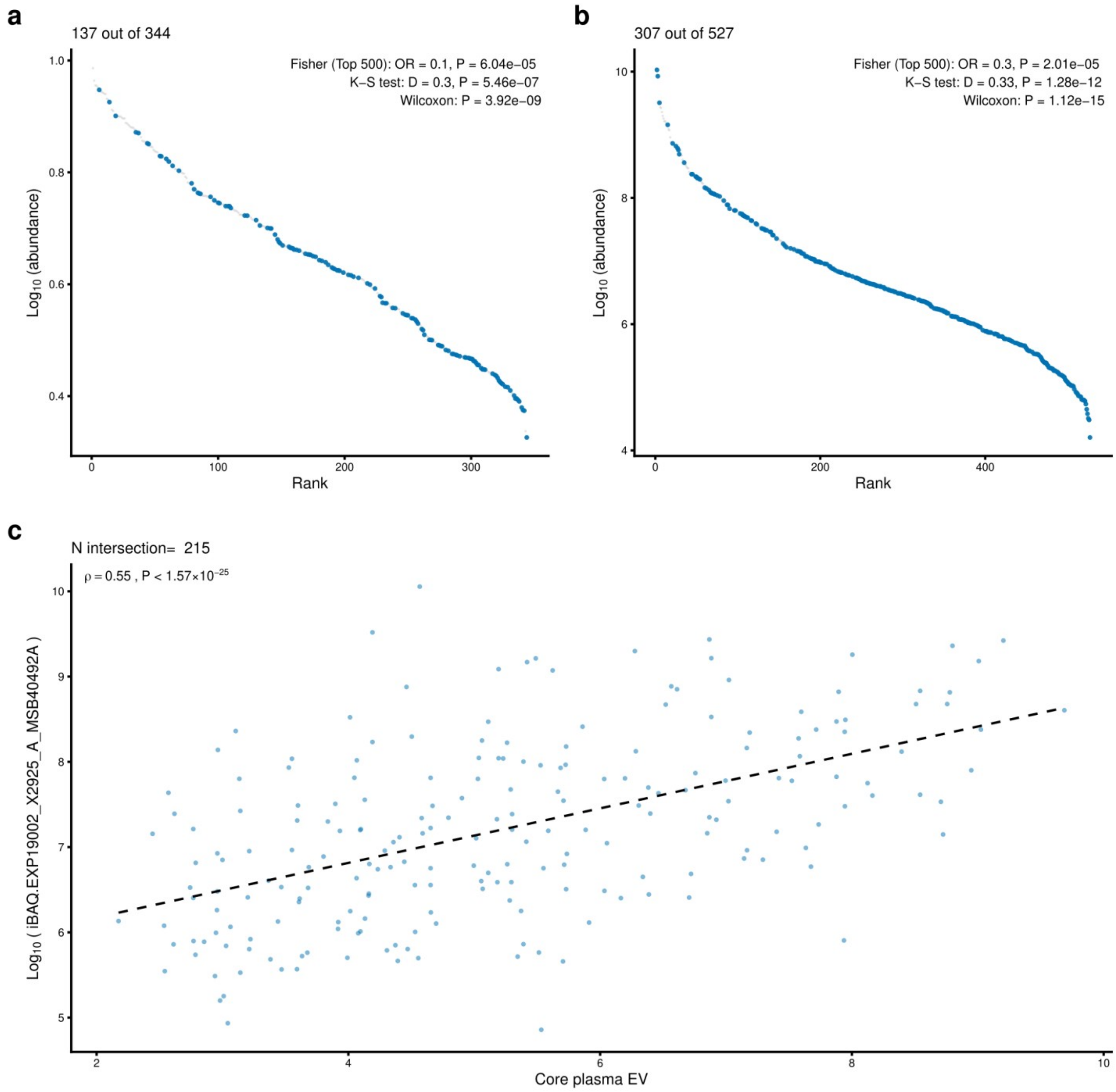

Fig. S8 | Stoichiometric alignment of the core plasma EV proteome and optimal synthetic nanoparticle corona. *(**a, b**) Rank-abundance profiles for the core plasma EV proteome and the highest correlating synthetic nanoparticle corona (Blume et al., sample X2925). The curves illustrate the concentration-driven distribution, with intersecting proteins highlighted in blue against the full dynamic range of each respective proteome (grey). The statistical significance of the top-ranking distribution overlap is evaluated via Fisher's exact test, Kolmogorov-Smirnov (K-S) test, and Wilcoxon rank-sum test. (**c**) Scatter plot comparing the quantitative* Log_{10}*- abundances of the N=215 intersecting proteins. A strong positive monotonic relationship is observed between the core plasma EV proteome and the X2925 nanoparticle corona, evaluated by Spearman's rank correlation (*$\rho=0.55$*,* $P=1.57\times10^{-25}$*). The dashed black line represents the linear regression fit. This compositional correlation supports a surface-charge-driven mass-action mechanism for plasma EV co-isolation.*

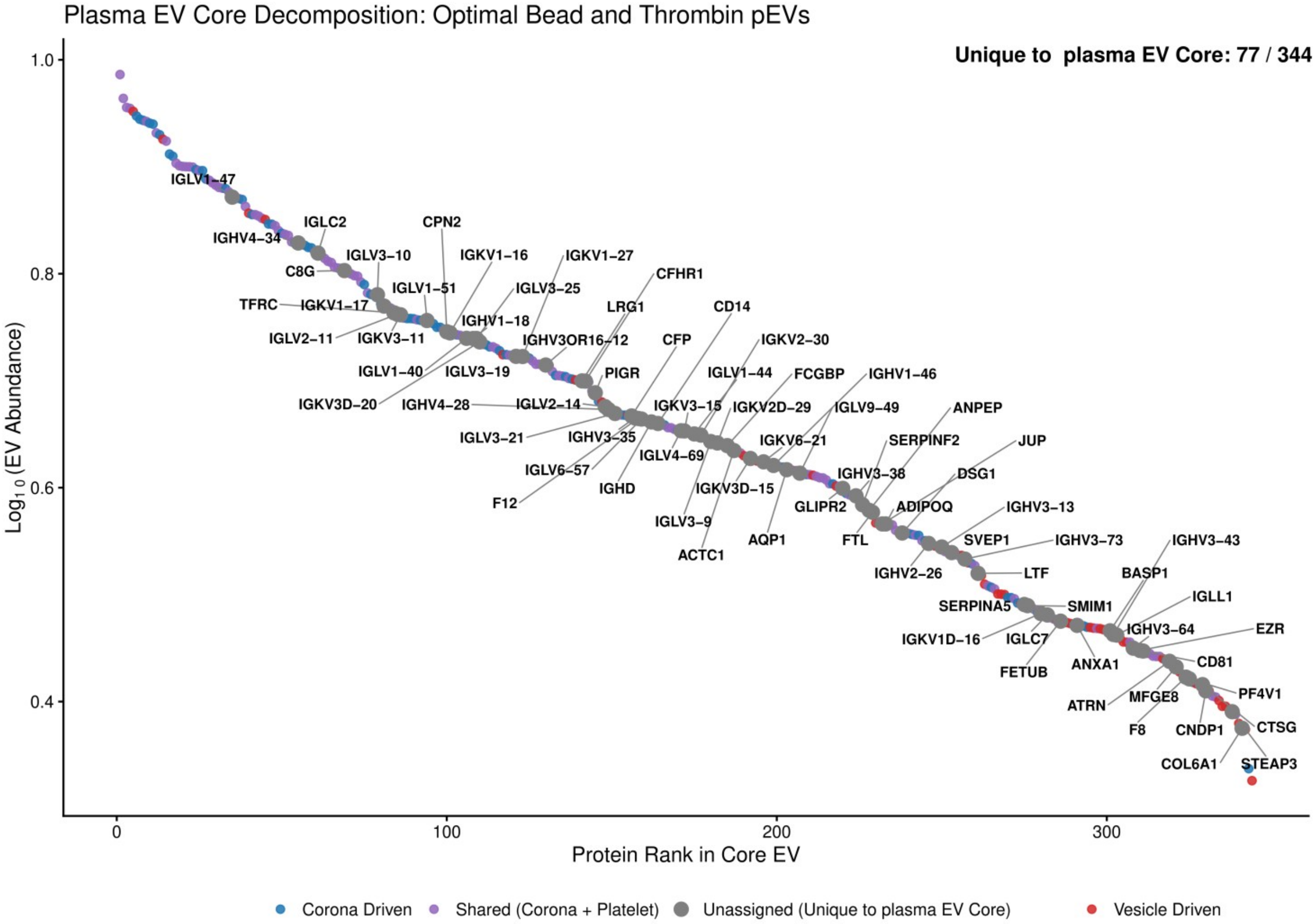

Fig. S9 | Quantitative deconstruction of the core plasma EV proteome using optimal biophysical and cellular reference models. *Rank-abundance distribution of the core plasma EV proteome (344), partitioned by quantitative intersection with the highest-correlating synthetic and cellular reference datasets. Protein origins are categorized as follows: Corona Driven (blue) represents proteins shared exclusively with the optimal synthetic nanoparticle corona (Blume et al., sample X2925, pI 5.8); Vesicle Driven (red) represents proteins shared exclusively with ex vivo thrombin-stimulated platelet-derived EVs (Moon et al.), the highest correlating cellular activation state; and Shared (purple) indicates proteins quantified in both optimal reference sets. Proteins lacking a confirmed quantitative abundance in either reference dataset are designated as Unassigned (grey, 77). The persistence of these 77 unassigned proteins highlights the limitations of utilizing a single optimal biophysical surface or a single cellular activation state to model the complex co-isolation dynamics of plasma EVs.*

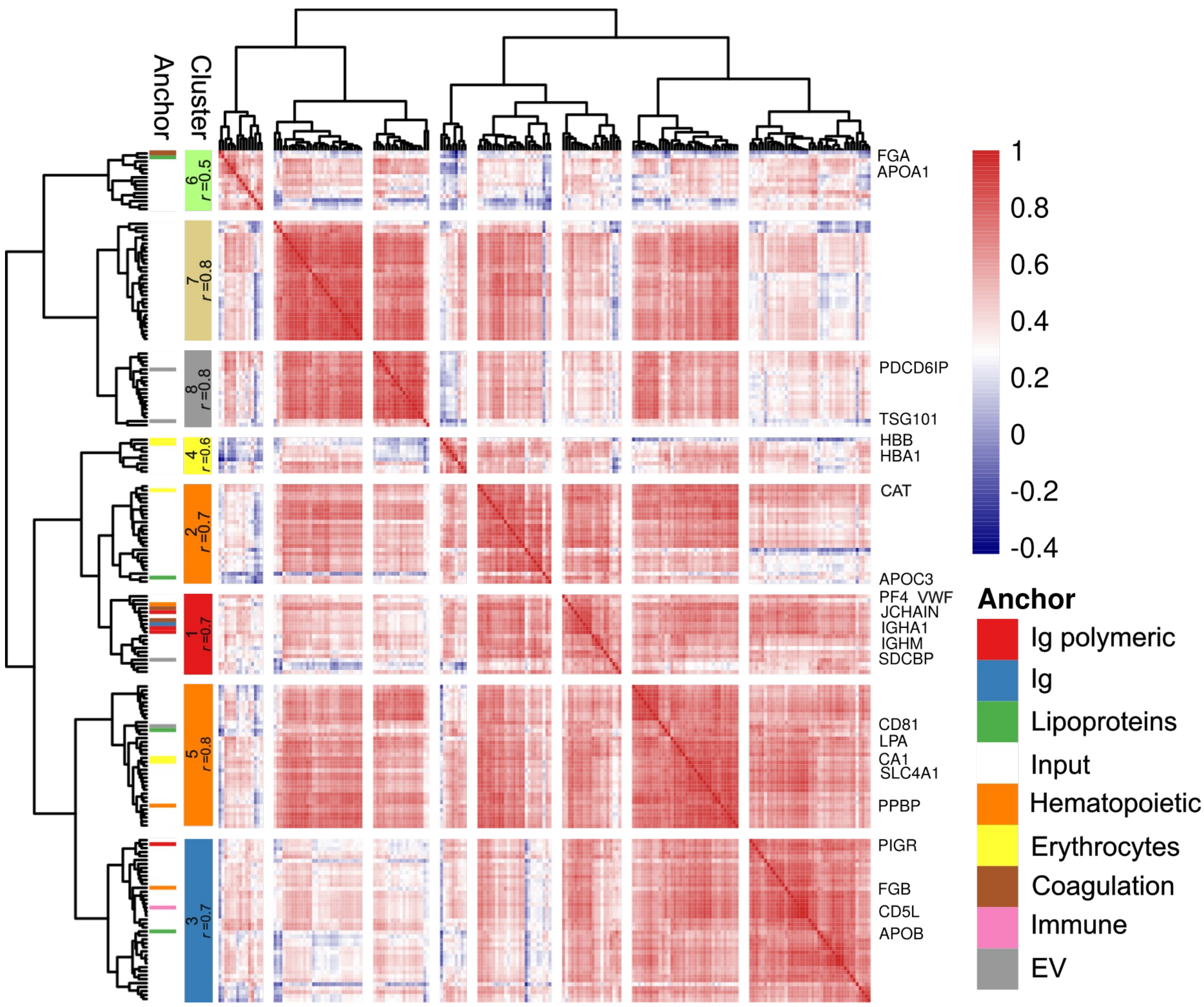

Fig. S10 | Application of the co-abundance framework to the Rai et al. candidate marker list and anchor proteins. *Unsupervised clustering of the candidate EV marker panel reported by Rai et al., performed using the same parameters as the core proteome analysis. Biological annotation and cluster color coding follow the exact scheme described in Fig. 7 and Table 1. The analysis resolves the consensus list into 8 distinct variance components, distinguishing vesicular markers from co-isolated plasma modules.*
