# Supplementary material for: Analysis of 515 human proteomes defines plasma extracellular vesicle core composition, corona and coisolated networks
Source: Commun Biol. 2026 Oct 30;9:1402. doi: 10.1038/s42003-026-11038-6 (PMC13722686; doi:10.1038/s42003-026-11038-6)
Supplement: Supplementary file 3 [file 42003_2026_11038_MOESM3_ESM.pdf]

Description of Additional Supplementary Files:

File: Supplementary Data 1

Description: Literature extracted protein markers for signatures expected to be present in plasma EVs.

File: Supplementary Data 2

Description: Rank-normalized expression values for EV core proteins.

File: Supplementary Data 3

Description: Conservative lists of protein signature markers utilized for module definition.

File: Supplementary Data 4

Description: Abundance-weighted isoelectric points (pI) of proteins identified from engineered synthetic nanoparticles in Blume et al.20 and in the plasma core EV proteome.

File: Supplementary Data 5

Description: Coverage-Weighted Spearman's rank correlation between engineered synthetic nanoparticles in Blume et al.20 and in the plasma core EV proteome.

File: Supplementary Data 6

Description: Coverage-Weighted Spearman's rank correlation between the plasma EV core and platelet-derived EVs (pEVs) stimulated by ADP, collagen, thrombin, and a thrombin/collagen dual-stimulation.

File: Supplementary Data 7

Description: Identified protein intersection between the single optimal synthetic corona (Blume20 Sample X2925) and the optimal platelet activation state (Thrombin pEVs).

File: Supplementary Data 8

Description: Identified protein intersection between the Pan-Corona (the maximal abundance across all 11 synthetic nanoparticle chemistries) and the Pan-Platelet (the maximal abundance across all agonist-induced pEV states).

File: Supplementary Data 9

Description: Proteins comprising the distinct co-variance clusters derived from the core plasma EV proteome.

File: Supplementary Data 10

Description: Proteins comprising the distinct co-variance clusters based on EV feature proteins reported by Rai et al.7
